# A Novel Analytical Strategy to Identify Fusion Transcripts between Repetitive Elements and Protein Coding-Exons Using RNA-Seq

**DOI:** 10.1371/journal.pone.0159028

**Published:** 2016-07-14

**Authors:** Tianyuan Wang, Janine H. Santos, Jian Feng, David C. Fargo, Li Shen, Gonzalo Riadi, Elizabeth Keeley, Zachary S. Rosh, Eric J. Nestler, Richard P. Woychik

**Affiliations:** 1 National Institute of Environmental Health Sciences (NIEHS), 111 TW Alexander Drive, Building 101, Research Triangle Park, NC, 27709, United States of America; 2 Fishberg Department of Neuroscience and Friedman Brain Institute, Icahn School of Medicine at Mount Sinai, One Gustave L. Levy Place, Box 1065, New York, NY, 10029, United States of America; 3 Centro de Bioinformática y Simulación Molecular (CBSM), Departamiento de Bioinformática, Facultad de Ingeniería, Universidad de Talca, Av. 2 Norte, 685, Talca, 3465548, Chile; University of South Carolina School of Medicine, UNITED STATES

## Abstract

Repetitive elements (REs) comprise 40–60% of the mammalian genome and have been shown to epigenetically influence the expression of genes through the formation of fusion transcript (FTs). We previously showed that an intracisternal A particle forms an FT with the *agouti* gene in mice, causing obesity/type 2 diabetes. To determine the frequency of FTs genome-wide, we developed a TopHat-Fusion-based analytical pipeline to identify FTs with high specificity. We applied it to an RNA-seq dataset from the nucleus accumbens (NAc) of mice repeatedly exposed to cocaine. Cocaine was previously shown to increase the expression of certain REs in this brain region. Using this pipeline that can be applied to single- or paired-end reads, we identified 438 genes expressing 813 different FTs in the NAc. Although all types of studied repeats were present in FTs, simple sequence repeats were underrepresented. Most importantly, reverse-transcription and quantitative PCR validated the expression of selected FTs in an independent cohort of animals, which also revealed that some FTs are the prominent isoforms expressed in the NAc by some genes. In other RNA-seq datasets, developmental expression as well as tissue specificity of some FTs differed from their corresponding non-fusion counterparts. Finally, *in silico* analysis predicted changes in the structure of proteins encoded by some FTs, potentially resulting in gain or loss of function. Collectively, these results indicate the robustness of our pipeline in detecting these new isoforms of genes, which we believe provides a valuable tool to aid in better understanding the broad role of REs in mammalian cellular biology.

## Introduction

Epigenetic modifications such as DNA methylation and post-translational changes of histone tails have been shown to influence the expression of protein coding regions across the genome. Interestingly, the approximately 5 million repetitive element (RE) loci, which collectively comprise 40–60% of the mammalian genome [1] are also under epigenetic control [2]. Most notably, several lines of evidence have indicated that REs may become actively transcribed under circumstances where epigenetic repression is diminished such as during early development [3], aging [4] and cancer [5].

While the full extent of the biological impact of expressing REs is still poorly understood, one known outcome is the ectopic regulation of adjacent protein-coding genes through the formation of FTs. For instance, earlier work involving the analysis of three independent alleles of the viable yellow *A*^*vy*^ *agouti* mouse mutant showed that an intracisternal A particle (IAP) upstream of the first coding exon of *agouti*, when epigenetically unsilenced, hijacked expression of the gene [6,7]. This occurred through the formation of a FT that initiated transcription from a cryptic promoter within the Long Terminal Repeat (LTR) of the IAP reading through the coding exons of *agouti*. Transcription from the IAP led to expression of the agouti protein ubiquitously throughout the animal, and resulted in a phenotype involving yellow fur, obesity and the development of type 2 diabetes by 8 weeks of age [6,7]. It was also notable that the penetrance of the phenotype, including the severity of obesity, was variable within littermates: some of the mice were completely yellow, some had variable degrees of mottled fur while some were phenotypically normal, i.e., pseudoagouti mice. This variable penetrance was shown to be correlated with the epigenetic silencing of the IAP through methylation [6,7]. The penetrance of the *A*^*vy*^ trait was later shown to be strongly influenced by the environment: a variety of compounds in the mother’s diet change the penetrance of *A*^*vy*^ in the offspring by affecting the methylation status of the IAP [8–10].

Although *agouti* was one of the first examples, the use of next generation (NextGen) sequencing tools has more recently revealed broad-based formation of FTs across the mammalian genome involving different types of repeats and genes [3,5,11–16]. Despite this fact, only a few of these FTs have been linked to a specific cellular phenotype [5,16,17]. Thorough understanding of the extent to which REs impact the biology of a cell through the formation of FTs requires a sensitive, accurate and more comprehensive genome-wide approach for identifying FTs, which has yet to be achieved. While some progress has been made for the identification of FTs genome-wide, there remains a high level of false positives (>50%) with these existing approaches [5,11].

To gain more insights into the frequency, abundance and expression pattern of FTs, here we describe the development of a sensitive and highly specific bioinformatics pipeline applied for the genome-wide detection of FTs using RNA-seq datasets. We applied this pipeline to the analysis of data generated from the nucleus accumbens (NAc), a key reward structure of the brain, of cocaine-treated mice [18,19]. Repeated cocaine exposure leads to decreases in the levels of the repressive histone 3 lysine 9 trimethylation (H3K9me3) epigenetic mark primarily over REs, which can lead to their transcriptional upregulation [18]. The mechanism to explain how and whether the epigenetically-mediated changes in RE expression contribute to the biological outcome of cocaine exposure is currently unknown. The hypothesis driving our work was that activation of REs in response to cocaine leads to the formation of FTs, some of which may contribute to the behavioral changes associated with cocaine. Here we show that hundreds of genes express FTs with protein coding exons in the NAc, with different types of repeats involved in the fusions. We went on to validate FT expression by reverse transcription PCR (RT-PCR) using RNA from an independent cohort of animals. Using both RNA-seq (from several datasets) and quantitative real time PCR (qRT-PCR), we found that some FTs differ in their level of expression, tissue specificity and developmental regulation from their non-fusion counterparts. Given the potential role of FTs in shaping the biology of the cell, our data point to the robustness of our pipeline and the importance and utility of including repeats in the analysis of the transcriptome.

## Materials and Methods

### Animals and cocaine administration

Adult male C57BL/6J mice (Jackson laboratory) approximately 8 weeks old were used in this study. Animals were habituated for at least 1 week before experimentation. They were housed 5 per cage on a 12-hr light-dark cycle at constant temperature (23°C) with access to food and water *ad libitum*. Animals were killed by decapitation without anesthesia or analgesia, since all known anesthetic and analgesic agents affect the neural circuits and their biochemical constituents that are under study. Such decapitation occurs within a second and is standard practice. The health of all animals is monitored at least once daily. All animal protocols were approved by the Institutional Animal Care and Use Committee of Mount Sinai. Animals received daily i.p. injections for 7 consecutive days of cocaine HCl (Sigma) at 20 mg/kg body weight. Mice were used 24 hr after the final injection. Control mice for all groups received saline injections. Bilateral 14-gauge NAc punches were taken from each animal.

### RNA isolation and RNA-seq

RNA isolation and RNA-seq were performed as described previously [19]. In brief, brain samples were homogenized in Trizol (Invitrogen) and processed according to the manufacturer’s instructions. RNA was purified with RNeasy Micro columns. Four μg of total RNA were used for mRNA library construction following instructions of Illumina mRNA sample preparation kit (cat# RS-100-0801). The poly-A containing mRNA was purified using poly-T oligo-attached magnetic beads. The mRNA was then fragmented into small pieces using divalent cations under elevated temperature. The cleaved RNA fragments were copied into first strand cDNA using reverse transcriptase and random primers. This was followed by second strand cDNA synthesis using DNA Polymerase I and RNaseH. These cDNA fragments went through an end repair process, the addition of a single ‘A’ base, and then ligated to the adapters. These products were gel purified and enriched with PCR to create the final cDNA libraries. The library constructs were run on the bioanalyzer to verify the size and concentration before sequencing on the Illumina HiSeq2000 machine at Mount Sinai’s Genomic Core facility. The raw RNA-seq reads were initially processed by trimming adaptor sequences from the 3’ end, filtering with average quality scores greater than 20 and length at least 25 bases. The reads passing the initial processing were aligned to the mouse reference genome (mm9; Genome Reference Consortium Mouse Build 37 from July 2007) using Bowtie or TopHat-Fusion.

### TopHat-Fusion Pipeline and Specific Identification of FTs

Fusion-detecting tool TopHat-Fusion version 2.0.4 was used for identification of FTs for each sample [20]. The following modifications to the default parameters were incorporated: “–g 1” (uniquely mapped), “–N 1” (allow one mismatch), “--max-intron-length 10”, “--max-segment-intron 10”, “--fusion-read-mismatches 1”, “--fusion-multireads 1” and “--fusion-anchor-length 25”. One of the output files from the software is fusions.out, which includes the list of fusion events supported by at least one read alignment. We developed Perl scripts to determine the identity of each section of the potential fusion. Chimeric transcripts assigned to repeat-repeat and to exon-exon were excluded and only those containing a repeat and an exon retained. At this step, information about genomic location, read count, exon and gene, repeat class and splicing site sequence were recorded. Next a read count filter of at least 2 reads crossing the exact junction site was applied, and only fusions in which each section was at least 25 base-long were further analyzed. The script was subsequently re-applied to update RE and exon annotations, genomic location and read count of each fusion; the uniqueness of the sequences was further checked. The list of fusion genes presented on S1 Table was obtained upon identification of at least one qualifying event per gene. A qualifying event was defined by the presence of a FT that had at least 2 supporting reads in 2 out of the 3 samples from the same group. Therefore, events having 1 read in one sample were considered only if the two other samples from that group had more than 2 reads supporting it. The scripts are available upon request (wangt4@niehs.nih.gov).

Additional publicly available RNA-seq data sets were used to determine the tissue- and stage-specificity of FT expression. They are from various brain regions (Nestler et al., unpublished), from other tissue types (Wellcome Trust Sanger Institute, http://www.sanger.ac.uk/resources/mouse/genomes/), as well as from different developmental stages including the oocyte and 2 cell-stage [3] and 6 stages of the cortical layer formation in the brain [21]. The number of reads that mapped over the junction of the repeat-exon (FT counts) to the reads that mapped over the corresponding exon-exon junction of the gene, involving the same exon, (non-fusion counts) was compared. Only the reads with at least 15bp from one side of the junction were counted.

### Hard Clipping Fusion Gene Candidate Estimation with Bowtie

Short read aligner Bowtie version 1.0.0 with minor modifications on the default settings (-v 1 and -m100K) was used to estimate the number of chimeric reads or fusion genes in the NAc transcriptome. REs were defined by RepeatMasker and downloaded from the University of California Santa Cruz (UCSC) Genome Browser (http://genome.ucsc.edu). Gene annotations were based on KnownGene, which was also downloaded from the UCSC Genome Browser. To identify the chimeric reads, the GenomicRanges package of R/BioConductor version 2.14 was used. Reads that did not map full-length to the genome and had 25 bases at one end derived from a repeat locus (not mapping within a defined exon) and the other 25 bases to an exon were considered fusion reads. Scripts were developed to check the uniqueness of the 25 bases aligning to the exon, which was then used to define the identity of the candidate fusion gene which was recorded. The estimated pool of candidate genes that express FTs was calculated based on the identified genes relative to the total number of annotated genes (~29K) on mm9.

### Clustering analysis

Unsupervised hierarchical clustering analysis was performed with Cluster 3.0 to group the samples with correlated counts [22]. The results were displayed as Heatmaps using TreeView [23].

### Estimation of Observed versus Expected Repeats in FTs

A Perl script was developed to count all possible FT combinations involving exons (genes) and their nearest REs using the gene model (.gff3) and RepeatMasker annotations from mm9. The frequencies were calculated per RE class (DNA, LTR, LINE, SINE, SSR and Low_complexity) based on the genomic region of the repeat (intronic or intergenic) from which start and end positions were recorded. Exons were used as anchors to define the nearest repeat, and the intronic or intergenic frequencies of each RE class summed. As the start and end positions of REs were recorded, statistics were run separately to confirm each other. The absolute frequency for each RE class is shown as the sum of these values per genome region, which as expected, was comparable. The script is available upon request to Dr. Gonzalo Riadi (University of Talca).

### Experimental Validation of Predicted FTs and qRT-PCR Analysis of Non-Fusion and FT Levels

For biological replication, a separate cohort of animals was prepared for cocaine i.p. injection and tissue collection. After isolation via RNeasy Micro columns (Qiagen), RNA was then reverse transcribed using a Bio-Rad iScript Kit. cDNA was quantified by quantitative PCR (qPCR) using SYBR Green (Quanta). Each reaction was run in duplicate or triplicate and analyzed following the ΔΔCt method as previously described using glyceraldehyde-3-phosphate dehydrogenase (GAPDH) or beta-2-microglobulin (B2M), which are not altered by cocaine exposure, as normalization control. The PCR products were verified on an agarose gel and Sanger sequencing was used to confirm the size and sequence accuracy. For comparisons of the levels of expression of non-fusion vs FT isoforms, primers were designed such as to amplify the RE and exon present in the FT, and the same exon but when spliced to the next non-fusion control locus, as shown in S2 Table.

### Statistical Analysis of Differentially Expressed FTs based on RNA-seq counts

As presented on S1 Table, we used the logarithm base 2 of fold change to identify a group of differentially expressed FTs from cocaine- with respect to saline-treated animals. Starting from the average read counts of the samples in each treatment, fold change was calculated as follows:

$${\text{log}}_{2}\left(Fold\;change\right)=log_{2}\left(\frac{{\langle read\;counts\rangle }_{cocaine\;samples}}{{\langle read\;counts\rangle }_{saline\;samples}}\right)$$

The flanking angle brackets in the formula represent the average function. Before applying the formula, we arbitrarily summed 1 read count to all samples that contained ‘0’ counts from both sample groups. The p-values were calculated for each event as the fraction of fusion events with a value larger or equal than the event’s logarithm of fold change. We used confidence level of 5% for the tests.

Additionally, for each FT, we applied the Wilcoxon sum of ranks test to the raw RNA-seq fusion counts comparing read counts from saline to cocaine for each event to obtain an observed test statistic score. To increase confidence of those findings, we also performed bootstrapping sampling (B = 10,000) with replacement. In brief, based on read counts from within each group we randomly sampled combinations of read counts 10,000X, which were then used to compare each fusion event between saline and cocaine. This generated a non-parametric distribution of test statistic scores. The *p*-value for a FT was determined as the number of times (n) that a test statistic score from the comparisons of the bootstrapped samples was > the positive observed test statistic score or < the negative observed test statistic score. Hence, *p*-value = n/B; the *p*-value of each event was calculated using R package version 2.14.1.

## Results

### Analytical Pipeline to Detect FTs with High Specificity Using TopHat-Fusion

**Fig 1:**
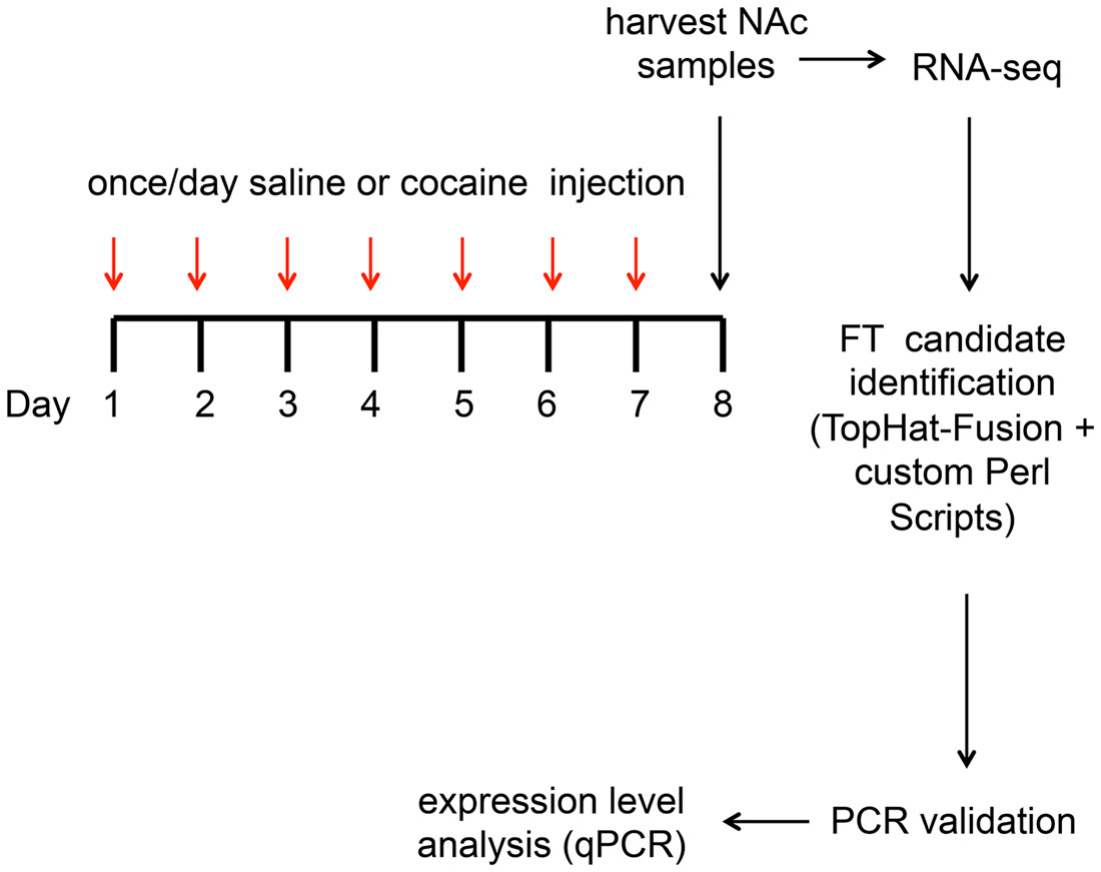
Diagram of experimental design and analysis. Male mice (15 per group) were treated once daily with either cocaine or saline, which were administered intra peritoneally (i.p), for 7 consecutive days. Twenty four hours after the last injection animals were sacrificed and the nucleus accumbens (NAc) harvested. RNA was then processed and submitted to RNA-seq (Illumina HiSeq). The resulting reads were analyzed through our modified TopHat-Fusion pipeline designed specifically for the identification of repeat-fusion transcripts (FTs). More detailed steps of the pipeline are presented in S1 Fig. Expression of candidate FTs were then first validated by reverse-transcription PCR in an independent cohort of animals, and expression level of FTs estimated using quantitative real-time PCR (qPCR).

To identify FTs with high specificity, we developed a bioinformatics pipeline centered around the software package TopHat-Fusion, which was created to identify chimeric transcripts arising from chromosomal translocations in cancer [20]. We reasoned that we could use this software as a starting point to identify fusion events, and develop new analytical methods to fine-tune the output for FT detection. Algorithmic details on this fine-tuning including the imposed filtering are found in Methods. Our pipeline, which can be applied to single- or paired-end reads, involves a series of stringent criteria including: (i) each end of the fusion had to be mapped uniquely (-m1), allowing one mismatch. This step eliminated ~10% of reads from the data (see below); (ii) one end of the fusion had to be mapped to a repeat locus and the other end had to map to an annotated exon, (iii) the junction site of the fusion had to be flanked by canonical GT/C-AG splicing dinucleotides, which eliminated false positives associated with unspliced or partially spliced transcripts and (iv) at least 2 reads supporting the fusion had to be detected in 2 out of the 3 independent replicates from one of the experimental groups. Note that the reads to support the fusion had to span the exact junction (splice) site. Therefore, the FT read count is much lower than the typical representation of RNA-seq counts and may not reflect the overall expression level of the FT. In this study the experimental groups were composed of a total of 15 saline- or 15 cocaine-exposed mice from which 3 independent replicates were created by pooling material from the dissected NAc of 5 individual animals. A diagram representing the experimental design and analysis is shown in Fig 1.

**Fig 2:**
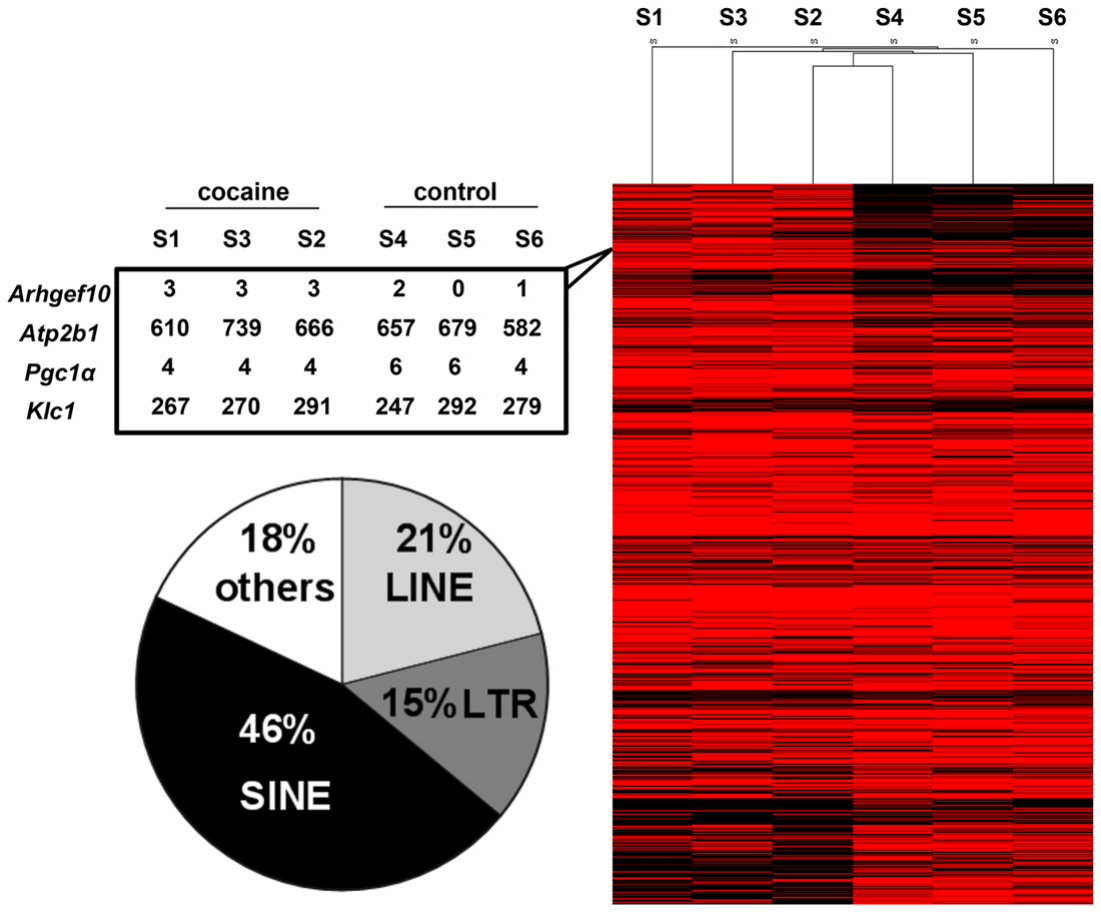
FTs are expressed in the NAc of saline- or cocaine-treated mice. (A) Heatmap represents the read counts of each fusion event identified in either the saline- or cocaine-treated samples; non-supervised hierarchical clustering was applied separating cocaine- from saline-exposed samples. Red indicates higher number of reads while black indicates lower number of reads. The table on the left shows examples of reads counts, which must span the exact fusion junction, supporting the fusions. S1–S3 and S4–S6 are the 3 independent samples exposed to cocaine or saline, respectively. Pie chart indicates the frequency of fusion events with different classes of repeat elements.

We studied all 4.9 million repeat loci including the most common classes of REs present on the mouse genome assembly (mm9): Long Interspersed Nuclear Elements (LINEs), Short Interspersed Nuclear Elements (SINEs), LTRs, DNA repeats and satellites. A summarized analytical workflow is shown in S1 Fig Details about the treatment of the animals, the RNA-seq and the pipeline are presented in Methods. This approach identified FTs expressed from ~800 genes from the saline- or cocaine-exposed samples that matched the first 3 stringent criteria, and 438 genes that had a qualifying fusion event present in all 3 samples from either group (see Methods for details). These 438 genes together expressed 813 different FTs, which were considered distinct since the genomic coordinates of the junction sites were not the same (S1 Table). The majority of FTs involved SINEs (46%), followed by LINEs, LTRs and others, respectively (Fig 2 and S3 Table). Given the genomic distributions of repeats within introns and intergenic regions and all the potential combinations to generate fusion events between these and the nearest protein coding exons, the FTs that involved simple sequence repeats (SSR) were significantly less frequent than expected (S3 Table).

### PCR validations of RNA-seq identified FTs

Read counts spanning the junction site of the fusions varied from 1–3 to 100s, depending on the gene (Fig 2 and S1 Table). Because of the low counts of many FTs (S1 Table), it was important to validate the TopHat-Fusion predictions using a different approach. Thus, we selected 20 out of the 438 genes expressing 28 different FTs to confirm the presence of the FT by RT-PCR. These 20 genes were prioritized based on biological function and known associations to cocaine addiction as well as their read counts (low and high); their identity and primer sequences are presented in S2 Table. We found that all 28 predicted FTs from the 20 selected genes gave rise to PCR products of the expected size; a representative gel is shown in S2 Fig. We then cloned and sequenced the RT-PCR products from 13 FTs to provide additional confirmation that they were derived from the genes of interest (data not show).

**Fig 3:**
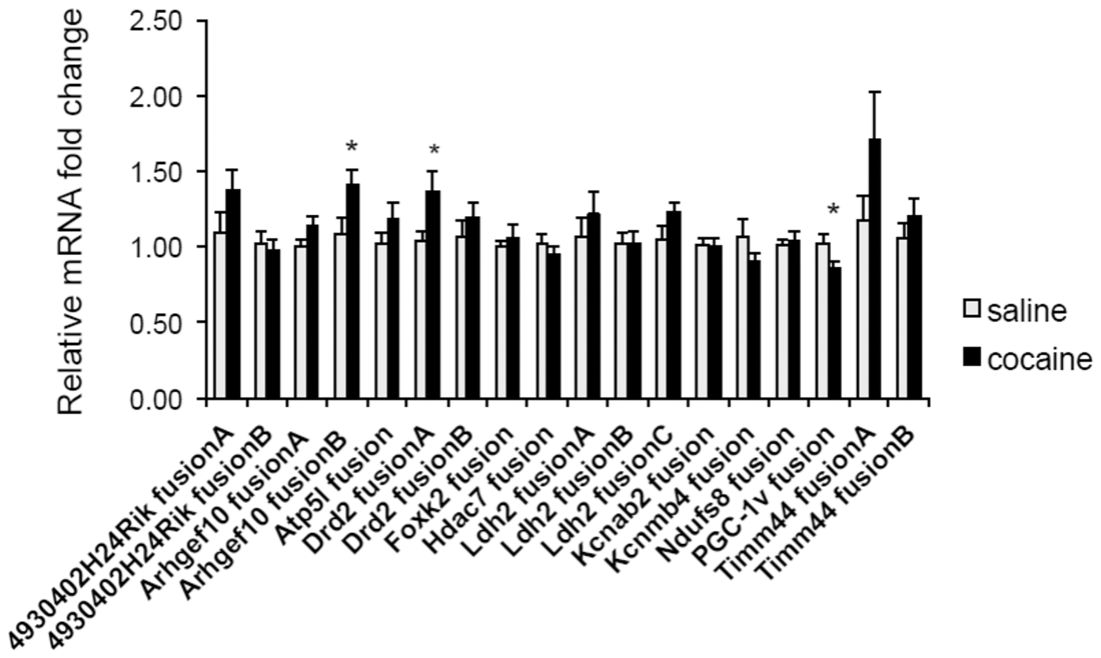
qRT-PCR validated the differential expression of some FTs. Relative fold changes to GAPDH of FT levels in the NAc 24 hours after seven daily cocaine i.p. injections, with saline injections as control. Data are presented as mean ± SEM. N = 10–12 per group. *p< 0.05, unpaired Student’s t test.

To determine whether any FT responded to the exposure to cocaine, we performed different statistical tests both on fusion read counts and fold-change, and confirmed the former by bootstrapping (sampling with replacement) the data. Bootstrap essentially repeatedly (in this case, 10,000 times) reassigns random values generated from the read count distribution within a group, populating a distribution of gene specific scores that overall increases the resolution of the test (for more details see Methods). The three statistical methods utilized revealed that 5–10% of the 813 FTs or about 10% of genes expressing FTs were differentially regulated (either up or down) upon exposure to cocaine (S1 Table). We then selected 12 genes (18 FTs), all previously shown to be involved with the effects of cocaine, to validate differential expression using qRT-PCR. Note that for these experiments RNA from an independent cohort of saline- and cocaine-treated animals were used. While 2 genes identified using the RNA-seq fusion counts were validated as differentially expressed (Fig 3), from the tested genes, the dopamine receptor 2 (*Drd2*) FT was not statistically different based on read counts but proved to be so by qRT-PCR (Fig 3). These findings highlight that fusion counts, especially those with low read numbers, do not necessarily reflect the level of expression of FTs (see also Discussion).

### Levels and Regulation of FT Expression

Having confirmed the specificity of the TopHat-Fusion pipeline in detecting these new gene isoforms, we next asked how the level of expression of the FT related to the expression of the non-fusion counterpart. To address this we performed qRT-PCR on 8 of the 20-PCR validated genes, which were prioritized based on counts and responsiveness to cocaine (S1 Table and Fig 3). We used primers that would uniquely compare the level of the FT with a non-fusion isoform of the gene that involved the same exon (S2 Table). Our results demonstrated that some genes produce high levels of FTs compared to the non-FT isoforms. For instance, the FTs for *Atp2b1* (the main membrane calcium transporter) and *Celf2* (CUGBP, Elav-like family member 2) were expressed at 40- and 10-fold higher levels, respectively, than their non-FT counterparts (Table 1). For the peroxisome proliferator-activated receptor gamma coactivator 1 alpha (*Pgc1α*), FTs were present at about equal levels to the corresponding non-FT isoform. In other instances (see Table 1), FTs were expressed at relatively low levels compared to their non-fusion counterparts. An extreme example is *Arhgef10* (Rho guanine nucleotide exchange factor 10), where the fusion A and B transcripts were expressed at levels that were less than 5% of the non-FTs from the gene.

**Table 1:** Fusion gene count and its fold expression relative to nearest control locus. The genes involved in the fusions are shown; for *Klc1* and *Arhgef10* the different fusion events are shown independently. Average read counts identified by RNA-seq in each of the 3 independent replicates from either saline or cocaine-treated animals is presented. The expression level of the fusion relative to the nearest control locus (same exon) for saline- and cocaine-exposed animals, respectively, was estimated by qRT-PCR using primers that amplified the repeat-exon junction or the junction between the same exon with the nearest canonical adjacent locus (exon). For more details see Methods. Average fold-change between saline- and cocaine- treated samples, and total standard error are shown; NA = not analyzed. In italic and bold are the 3 genes that are expressed at similar or higher levels than the non-fusion counterpart. Last column represents the results as the ratio of non-fusion over fusion gene expression; i.e. for every 1 transcript of non-fusion *Atp2b1* there are 40 transcripts expressing the fusion isoform.

	Mean fusion gene count		Fold expression relative to nearest control locus				Wild-type/fusion ratio
*Fusion gene*	saline	cocaine	saline	cocaine	total mean	total se	
***Atp2b1***	595.3	621.3	40.006	38.952	**39.479**	1.495	1:40
***Klc1 fusion A***	64.7	58.3	0.274	0.273	0.273	0.007	4:1
***Klc1 fusion B***	117.0	127.0	0.325	0.333	0.329	0.007	3:1
***Klc1 fusion C***	276.0	269.3	0.524	0.526	0.525	0.015	2:1
***Elfn2***	31.0	28.0	0.058	0.053	0.056	0.002	18:1
***Macf***	21.3	28.0	0.528	0.541	0.535	0.012	2:1
***Celf2***	155.3	157.0	10.527	10.408	**10.467**	0.340	1:10
***Srrm2***	44.0	50.3	0.149	0.144	0.146	0.006	7:1
***Pgc1α***	5.3	4.0	1.187	1.155	**1.171**	0.038	1:1
***Arhgef10 fusion A***	1.0	3.0	0.024	NA	0.024	NA	42:1
***Arhgef10 fusion B***	0.0	0.7	0.007	NA	0.007	NA	143:1

**Fig 4:**
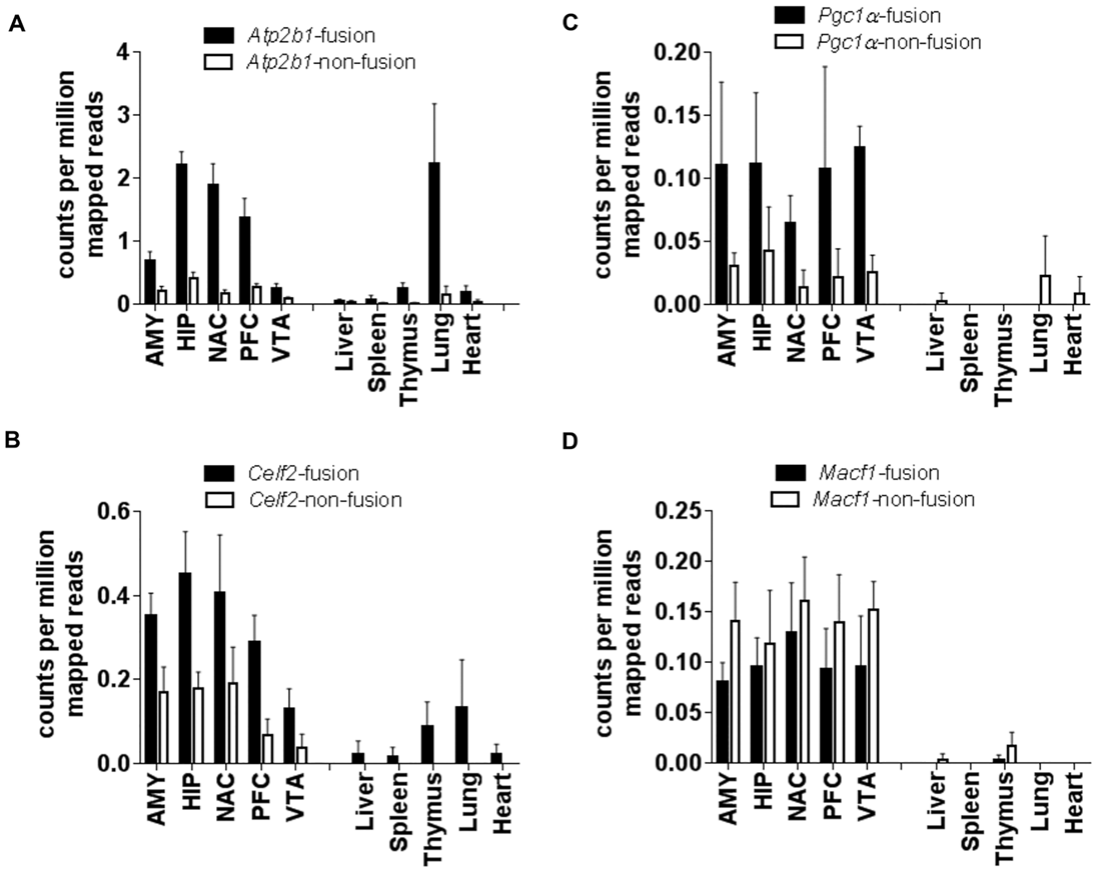
Some genes expressing FTs are expressed in several tissues while others are expressed in a tissue-specific manner. Read counts were compared between FTs and non-fusion isoforms of genes in different areas of the brain (left side of graph), including the amygdala (AMY), hippocampus (HIP), NAc, prefrontal cortex (PFC) and the ventral tegmental area (VTA) as well as in different tissues (right side of graph). Black bars represent average counts per million aligned reads ± SD from 4–6 independent RNA-seq samples of the fusion version of a transcript while white bars indicate the average ± SD for the non-fusion counterpart.

Subsequently, we interrogated whether any of the 8 FTs studied showed tissue- and developmental stage-specificity in their mode of expression. For this purpose we examined our own RNA-seq data from different sections of the brain (amygdala, hippocampus, NAc, prefrontal cortex and ventral tegmental area) and publicly available datasets from various tissues (lung, heart, liver, thymus and spleen) as well as from different developmental stages (see below). We compared the number of reads that mapped over the junction of the repeat-exon (FT counts) to the reads that mapped over the corresponding exon-exon junction of the same gene (non-FT counts). Analysis of *Atp2b1*, *Celf2*, *Klc1* (Kinesin light chain 1) and *Srrm2* (serine/arginine repetitive matrix 2) revealed that FTs from these genes were expressed in all tissues analyzed (Fig 4 and S3 Fig); levels of *Arhgef10* FTs were low in all tissues studied (S3 Fig). Conversely, expression of FTs from *Pgc1α*, *Macf1* (microtubule-actin cross-linking factor 1) and *Elfn2* (extracellular leucine-rich repeat and fibronectin type III domain containing 2) were clearly tissue specific with FTs found in the brain but not in any other tissue analyzed (Fig 4 and S3 Fig).

**Fig 5:**
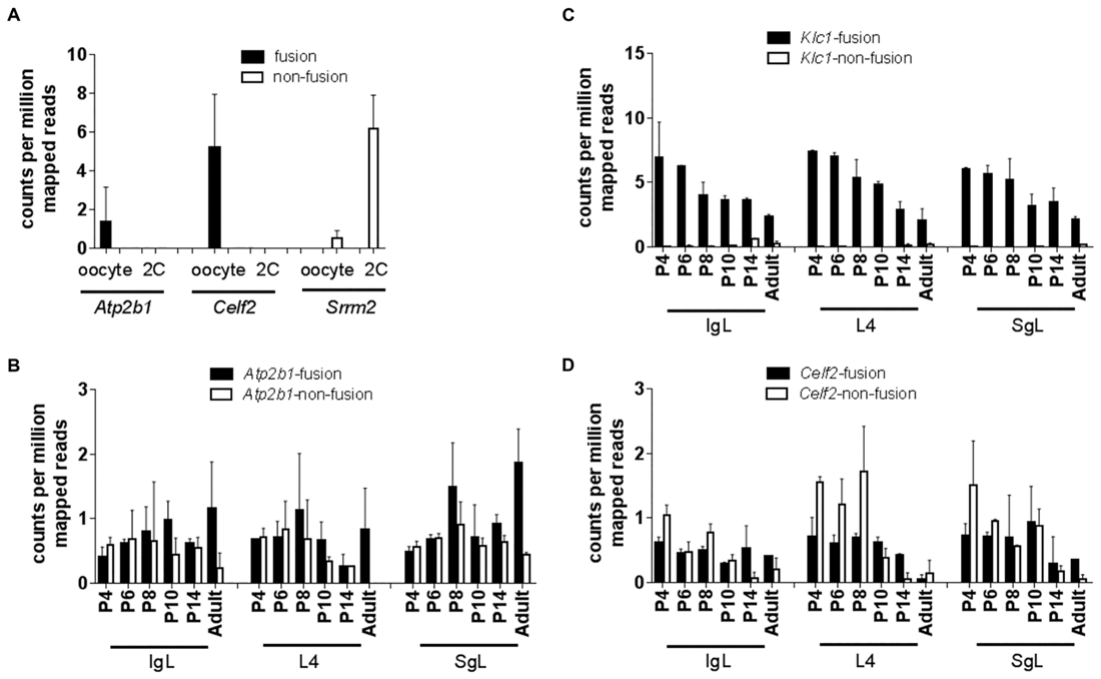
Regulation of fusion transcript expression differs from the non-fusion counterpart during development. Read counts were compared between FTs and non-fusion isoforms of the genes expressing FTs studied. Black bars represent fusion version of a transcript while white bars indicate the non-fusion counterpart. (A) Counts of the only 3 genes found to be expressed in the Macfarlan (2012) data set. Bars indicate the average read counts ± SD from 3 independent samples for oocyte or 2-cell (2C) stage, respectively. (B-D) Pattern of expression of fusion or non-fusion isoforms from 3 genes as a function of neocortex development; data for the other 5 genes can be found in Suppl. Fig 4. Bars represent average read counts ± SE from 2 samples (1 data set from males and other data set from female mice); thus statistical significance could not be tested. Data from 3 different layers were analyzed: IgL (infragranular layer), L4 (granular layer) and SgL (supragranular layer). P4–14 represent post-natal days 4 to 14.

To determine developmental or stage-specific regulation, we first analyzed the dataset from the oocyte and 2 cell-stage [3], and later mined data from cortical layer formation in the brain [21]. The results show an interesting pattern of developmental expression of FTs. Only three genes were expressed in the oocyte and 2 cell-stage: *Atp2b1*, *Celf2* and *Srrm2*. Their expression differed significantly from the expression of their corresponding non-FT isoforms (Fig 5A). For example, FTs from *Atp2b1* and *Celf2* were detected in the oocyte but not in the 2-cell stage embryo, while expression of the non-FT isoforms was not identified in these stages. Conversely, only the non-FT isoform of *Srrm2* was detected both in the oocyte and the 2-cell embryo (Fig 5A).

The formation of cortical layers in the brain occurs in an inside out manner involving essentially three layers: the infragranular layers L5/6 (IgLs), the granular layer (L4) and the superficial L2/3 or supragranular layers (SgLs) [21]. We analyzed RNA-seq from these different layers of the neocortex [21], which effectively served as independent replicates. We also analyzed different stages of development, including postnatal day 4 (P4), P6, P8, P10, P14 and P180 (adult). We found that the FT for *Atp2b1* was expressed at low levels, similar to its non-FT counterpart, at early stages of development and increased at P8. Expression increased ~2-5-fold while the corresponding non-fusion isoform decreased in expression in the adult brain (Fig 5B). The reverse was observed with *Klc1*, where the FT was more abundant in early development (P4–P10) and decreased ~5-fold in the adult; expression of the non-fusion isoform of *Klc1* only became apparent at later stages (Fig 5C). For *Celf2*, both fusion and the corresponding non-FT isoforms were identified at similar levels but decreased as the brain matured (P14–180). The non-FT isoform generally appeared to be more abundantly expressed at early developmental stages (Fig 5D). The pattern of expression of the other 5 genes, including *Arhgef10*, can be found in S4 Fig. Taken together these results suggest that the expression of FTs can be differentially regulated from the non-fusion counterpart in a cell/tissue-type and developmental-stage-specific manner.

### *In Silico* Reconstructions of Genes Giving Rise to FTs

**Fig 6:**
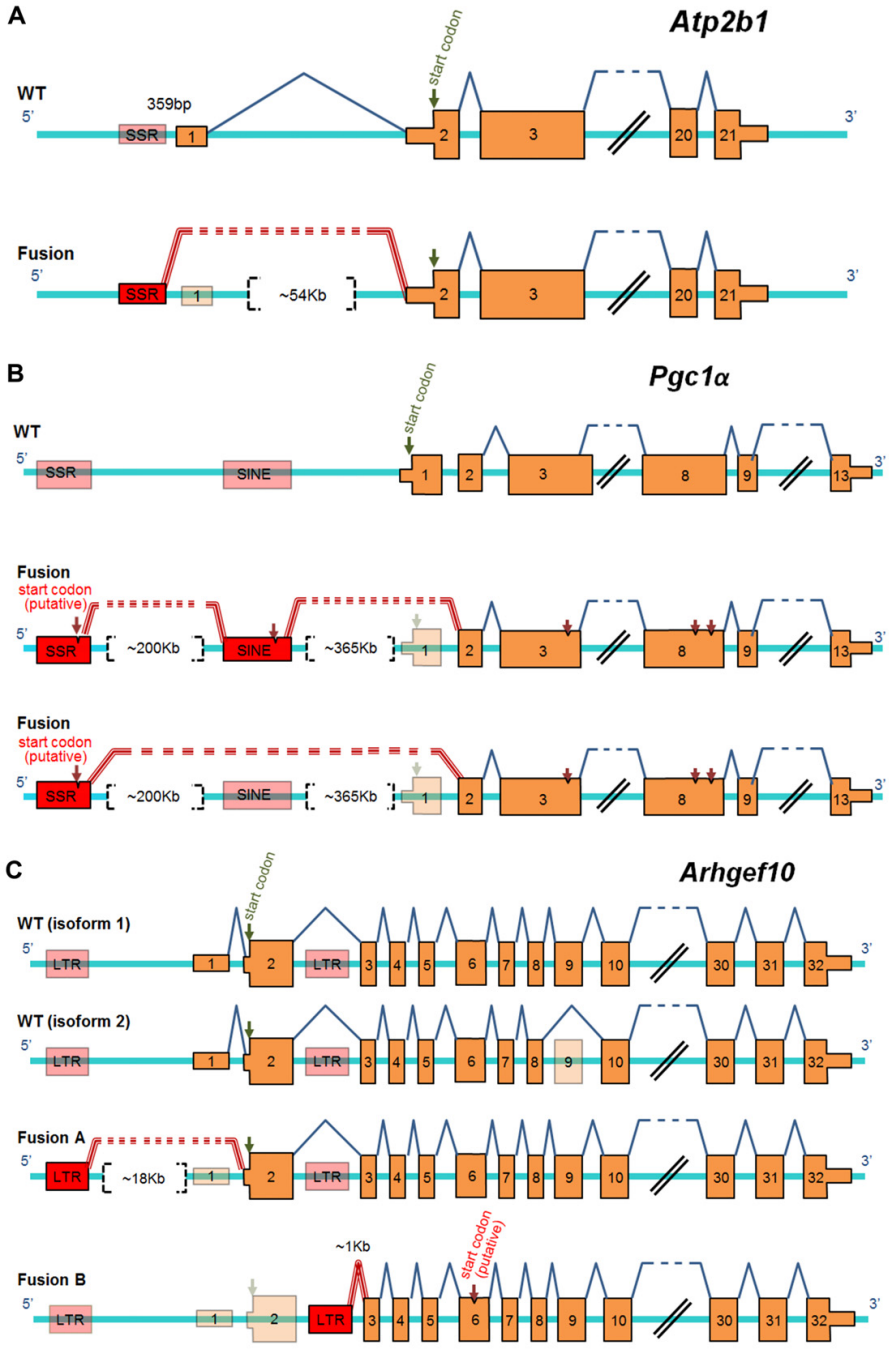
Representative diagrams of architecture of FTs. Diagram of the fusion events predicted in 3 genes: (A) *Atp2b1*, (B) *Pgc1α* and (C) *Arhgef10* where two isoforms of the non-fusion gene (including or not exon 9) are shown. Orange boxes represent annotated expressed (solid) or not expressed (transparent) exons in fusions; down arrows indicate translational start codons; putative TSS (transcriptional start site), start or stop codons are indicated. Red boxes represent the repeat involved in the fusion; blue lines above exons indicate exon-exon splicing while red triple lines represent exon-RE splicing. A double black line when used indicates not all exons of a given gene were depicted. Distances between repeat and exons are indicated in each figure.

We next asked about the impact of the FT to either gene expression regulation or to protein structure. To this end, we used the sequence of the fusion reads to reconstruct the genomic configurations that likely give rise to the FTs. We used the annotated intron-exon (KnownGene) and repeat coordinates on mm9 of 27 selected genes that produced at least one FT. From these analyses we determined not only that all of the different classes of REs studied can generate FTs but also that REs can function as alternative promoters, become new exons and/or lead to truncation of proteins. The genomic configuration of three genes giving rise to FTs illustrating the different ways that a RE can influence the expression of the gene through the formation of FTs is shown in Fig 6; reconstructions of 24 additional genes are shown in S5 Fig.

The first gene in Fig 6 shows the genomic configuration of a FT for *Atp2b1* (panel A). *Atp2b1* is a gene that is functional in a broad spectrum of cell types. The FT from *Atp2b1* involves a SSR 359 bases upstream from the annotated transcriptional start site (TSS). The SSR appears to function as an alternative promoter, forming a new transcript that splices to the first coding exon of the gene containing the annotated translation start site, which is about 54Kb away. Our ChIP-seq analysis of the NAc from saline- and cocaine-treated animals [19] revealed that the epigenetic marks H3K4me3 and H3K27ac, normally associated with promoters, map to this SSR (S6 Fig). Interestingly, the SSR associated with the FT in this gene is conserved throughout mammalian evolution (S7 Fig). Hence, similar to what was previously found with the FT associated with *agouti* [6,7], the FT for *Atp2b1* involves a RE likely functioning as an alternative promoter that regulates expression of a wild-type protein. Inspection of available mouse ESTs (expressed-sequence tags) at the National Center for Biotechnology Information (NCBI) confirm the presence of isoforms containing a fragment that maps to the SSR coordinates.

The second gene that we describe in Fig 6 is *Pgc1α*. *Pgc1α* derives its name from its interaction with the peroxisome proliferator-activated receptor gamma (PPARγ) and co-activates specific downstream targets of this nuclear receptor within the cell. The FT that we first identified for this gene involves a portion of a SINE, located about 365Kb upstream of the annotated promoter that is spliced to the second coding exon (Fig 6B). Additional analysis of the RNA-seq data revealed another fusion read that connects this SINE with a SSR located ~200Kb upstream from it. No additional RNA-seq reads upstream of (or connecting to) the SSR were identified. Thus we predict that the SSR corresponds to an alternative promoter region of the gene. We did, however, identify fusion reads that connect the SSR directly to the second coding exon of *Pgc1α*, excluding the SINE (Fig 6B). Both the SINE and the SSR are conserved between humans and mouse (S8 Fig), and have been previously reported as ESTs (NCBI). Given that the two different splicing events involving the SSR and SINE connect to the second coding exon of *Pgc1α*, which is downstream from the annotated ATG, the N-terminus of the proteins that we predict to be expressed from these FTs will differ from those expressed from the annotated non-FT counterparts for this gene. Therefore, *Pgc1α* is an example of a gene that gives rise to FTs that are predicted to change the structure of the expressed protein.

The third example shown in Fig 6 is *Arhgef10*, which is a gene that expresses FTs from two distinct LTRs with different DNA sequences. *Arhgef10* encodes a rho guanine nucleotide exchange factor that ultimately regulates the actin cytoskeleton in a way that can influence cellular morphology, migration and cytokinesis [24]. While both LTRs appear to be functioning as alternative promoters, the predicted protein derived from each FT is distinct. For instance, the first FT, fusion A, involves an LTR of an Endogenous Retroviral (ERV) family that is located ~18Kb upstream from the canonical TSS and that splices to the first coding exon of the gene (Fig 6C, upper panel). The second FT, fusion B, involves a distinct ERV located within the second intron that splices to the third exon of *Arhgef10*, which is downstream from the annotated ATG (Fig 6C, lower panel). While we predict that the fusion A transcript gives rise to the annotated normal protein, fusion B would need to recruit a downstream ATG in order to initiate translation. The first in-frame downstream ATG is located in exon 6, which, if recruited, would encode an N-terminal truncated protein that is about 206 amino acids shorter than the annotated non-fusion counterpart. Therefore, *Arhgef10* is an example of a gene that gives rise to two different FTs, one of which has the potential to encode a normal protein while the other is predicted to form a protein with an amino-terminal truncation.

Overall, reconstructing the genomic configurations of these 27 genes revealed that FTs have the potential to not only provide alternative promoters (similar to *agouti*), but, most notably, to potentially alter the structure of the protein being produced from that gene by either adding amino acids or skipping exons.

### Broad Estimation of Transcriptome-wide Fusion Gene Candidates Using Bowtie and Multiple Mapped Reads

The data presented attest for the robustness of the TopHat-Fusion pipeline in identifying FTs with high specificity. However, the 438 genes that we identified are likely to be an underestimate given that we limited the analysis to only reads that mapped uniquely on the genome, excluding those that were homologous to more than one site. While we attempted to use TopHat-Fusion to allow reads to map multiple times (-m250) across mm9, the computational demands of the software proved beyond the memory capacity of our servers (~4TB). Additionally, our requirement that the RE be spliced to a protein coding exon, which was necessary to exclude reads derived from primary unspliced transcripts (e.g., with RE sequences within introns), unfortunately also filtered out reads from FTs arising as contiguous fusion events with the adjacent genomic sequences, similar to the transcripts from the *A*^*vy*^ *agouti* allele. Finally, our pipeline included only those genes that had at least one qualifying event, that is, a FT present in all 3 independent samples from either the cocaine or saline treated groups.

Given these limitations, we sought an alternative approach using the software program Bowtie to better estimate the number of genes that may give rise to FTs across the genome. This software is not designed to detect FTs but is computationally efficient to map reads multiple times. The concept underlying this approach was to focus on reads that did not map full length, as they would likely be chimeras, and hard clip 25 nucleotides from each end of the read. We would then map each clipped section independently to mm9, and analyze those reads in which one end mapped to a RE while the other mapped to a protein coding exon. By anchoring the analysis on the exons we could then identify from which gene the fusion candidate was derived.

Implementation of the hard clipping analysis started by mapping reads from our saline and cocaine RNA-seq datasets to up to 100K loci (-m100K) to mm9. From the initial pool of ~96M reads in each dataset, we found that ~25M reads did not fully map to the reference genome (Table 2). Next, we independently mapped 25 bases at both the 5’- and 3’-ends of each read, and used RepeatMasker defined loci to determine whether either end aligned to a repeat. From this pool, we identified those reads where the opposite end mapped to an exon; reads that had both ends aligning to REs were discarded. We also excluded those reads where the end with the 25 bases aligning to an exon mapped more than once to the genome. Finally, we eliminated any reads that mapped to the mitochondrial DNA (mtDNA); this genome contains 37 genes (13 protein coding, 22 tRNAs and 2 rRNAs) but no introns. Genes that code for proteins are separated on the mtDNA by the presence of tRNA-encoding genes, which upon transcription of a polycistronic message are clipped off to release individual transcripts. Thus, it is not unexpected that “chimeric transcripts” would be identified from the mtDNA.

**Table 2:** Summary of hard clipping analysis for broad estimation of transcriptome-wide fusion gene candidates. The sequential steps taken and number of reads complying with each filtering step, per sample, are presented. S1–3 are the independent cocaine-exposed samples, S4–6 represent saline-treated animals.

	Cocaine			Control		
Single-end sequencing of RNAseq	S1	S2	S3	S4	S5	S6
Total number of reads	**96,974,552**	**96,520,741**	**95,144,928**	**97,494,354**	**97,535,022**	**93,184,881**
Trimming & QC filtering	96,409,441	95,968,626	94,646,921	96,948,185	96,893,189	92,665,973
Trimming & QC filtering (%)	99.42%	99.43%	99.48%	99.44%	99.34%	99.44%
**Full length mapped reads**	70,589,638	70,766,551	69,280,801	69,895,057	70,953,298	66,767,135
**Reads not full length mapped**	**24,996,271**	**24,396,311**	**24,682,246**	**26,201,363**	**25,061,110**	**25,183,673**
25bp of both 5' and 3' mapped	12,175,124	12,160,330	12,550,049	12,308,076	12,777,025	12,685,790
25bp of 5' or 3' mapped to REs (RepeatMasker)	972,039	915,441	947,438	955,282	840,227	1,069,409
One end mapped to REs, the other end mapped to non-repeat	317,240	310,698	314,339	322,885	286,541	321,566
**5' RE + 3' Exon (unique)**	**32,169**	**31,684**	**32,602**	**33,209**	**26,487**	**30,962**
**5' Exon (unique) + 3' RE**	**34,641**	**34,434**	**35,178**	**33,078**	**29,523**	**33,605**
**Estimated fusion genes**						
At least 2 reads per fusion event	6,898	6,881	6,933	7,084	6,210	6,888
Fusion event present in all 3 samples from each group		4,780			4,561	
Fusion genes identified in all 3 samples from either group		**5,553**				
**% of NAc transcriptome transcribed as fusion genes**		**19%**				
(Total 29,922 genes annotated in mm9)						

This simple strategy utilizing hard clipping identified an average of 5,553 candidate genes expressing FTs from either the cocaine or saline groups (S4 Table). About 87% of the genes identified by TopHat-Fusion as expressing FTs were also identified by this approach (S4 Table). The other 57 genes (~13%) involved fusions with low complexity repeat or other classes that were not examined. While we have not performed RT-PCR validation of the fusion events identified with this approach, cross comparison between the FTs detected by the TopHat-Fusion pipeline in *Arhgef10*, *Drd2 and Pgc1α* indicated that hard clipping identified the same events. Additionally, using the chromosomal coordinates of the 25 bases aligning to the RE and the exon we reconstructed 3 events only detected by hard clipping that involved repeats mapping more than once. We found that while repeats mapped to several locations, including in different chromosomes, in all cases there was at least one RE on the same chromosome and in close proximity (up to 5Kb) to the identified exon (data not shown). While not comprehensive, we conclude with these limited analyses that the hard clipping strategy may be informative in estimating broadly the number of potential FT-expressing genes. Currently, this number suggests that ~18% of genes across the mouse genome are likely to express FTs.

## Discussion

It is becoming increasingly clear that REs can have a substantial impact on the transcriptome of mammalian cells. In fact, most recently different analytical pipelines have been developed to analyze RE expression [25–29]. However, what is still not fully understood is the overall biological impact of actively transcribing REs. In this study we focused on better understanding the formation, expression and regulation of RE-associated FTs genome-wide. We started by identifying with high stringency the number of genes that express FTs in the cells using a TopHat-Fusion pipeline, which was followed by a parallel hard clipping strategy to get a broader estimate of the extent of genes that may express FTs in a cell. By developing a robust pipeline geared toward accurately identifying FTs across mammalian genomes using our TopHat-Fusion approach, we uncovered that: (i) hundreds of genes express FTs in the NAc of mice, (ii) all types of REs studied have the potential to form FTs, (iii) some FTs are the most abundant expressed isoform of the gene, (iv) FTs differ from their non-fusion counterparts in terms of tissue and developmental regulation, (v) some FTs may respond to the environment (in this study, cocaine treatment) and (vi) some FTs isoforms of may have the capacity to alter the structure of proteins expressed from a gene. Collectively, our results provide new insights into the outcome of active RE transcription as it relates to the formation of FTs with adjacent genes.

Previous work has demonstrated that epigenetically activated REs have two major biological outcomes. The first is the ability to retrotranspose, for those elements that have retained this capacity such as LINEs and SINEs, which may ultimately cause new insertional mutations. While this effect can be detrimental, as in cancer [30], others have indicated it may be part of the normal developmental program of an organism [31]. The second outcome is the ability to function as regulatory units of nearby or even distant genes through the formation of FTs [32]. While there have been many references to the formation of FTs in several cell types, full understanding of the biological significance of FT expression is still lacking. One of the reasons is that accurate FT identification remains challenging when mapping RE-derived reads from RNA-seq data that map to multiple loci. In addition, many individual reads that appear as fusion events between a RE and an adjacent non-repetitive sequence may be derived from unspliced transcripts from the nucleus of the cell. The TopHat-Fusion workflow used in this study established very stringent criteria for the identification of FTs genome-wide, including unique mapping of reads and splicing of the RE to a downstream exon, which proved highly accurate and eliminated fusion reads derived from primary unspliced transcripts. Indeed, 20 out of 20 genes expressing FTs selected to be validated were confirmed by PCR. However, the draw back to this approach was the inability to include reads that map to multiple loci and to identify read-through events that do not require splicing. Because of these limitations, the number/abundance of genes giving rise to FTs identified by the TopHat-Fusion pipeline is underestimated. The hard clipping analysis allowed a broader understanding of the extent of genes that are expressed as fusions in the NAc (5,553) by including multiple map reads. However, its relaxed inclusion criteria (for instance, no requirement for the presence of splice sites on the read) may also increase the rate of false-positives. More detailed analysis of the fusion events identified with hard clipping will be necessary to test the level of true-positives predicted by this approach. Irrespective of the exact number, our results support those from other investigators [3,11–13] that many genes across the mammalian genome express FTs.

By including the most frequent classes of REs in our analysis, we determined that every class has the ability to give rise to FTs, with SSRs, in addition to LTRs, frequently functioning as alternative promoters. Even though it can be argued that the base composition of some SSRs suggests that they may be CpG islands associated with adjacent promoter regions (fully annotated or not), our data indicate that this may not always be the case. For instance, the SSR from the *Pgc1α* fusion is ~565Kb away from the annotated TSS of the gene, and the SSRs involved in the *Celf2* fusions are not only intronic but about 13Kb away from the closest annotated TSS (depending on the isoform of the gene; S5 Fig). Thus, at least in some circumstances SSRs may function as independent alternative promoters. Exonizations of repeats, particularly SINEs, have been previously reported in humans and mice with an estimated rate of less than 1% [33]. Many SINEs are located within introns of genes but why some of these are spliced into mRNAs and function like exons while others are not is poorly understood. In fact, given the sequence similarities of many REs of the same type and the abundant distribution of different types of REs within and between genes, it is still puzzling that one repeat is recruited to produce a FT while other REs nearby, even of the same class, are not. Hopefully the analytical pipeline described in this report will serve as a tool to identify classes of REs that can create FTs and can eventually help define the ‘rules’ governing the formation of these novel gene isoforms. Ultimately, it would be ideal to have access to a pipeline that can accurately identify all FTs, including those that involve contiguous (read through) events with adjacent genomic sequences.

Our data also reveal that there are several mechanisms through which REs can influence the expression of a gene. As previously shown by us and others [3,6,16], we found that repeats in FTs can work as alternative promoters (e.g. *Atp2b1* and *Arhgef10*). They can also be included as new coding exons that may change protein sequence and presumably function (e.g. *Pgc1α* and *Klc1*) and, most interestingly, potentially give rise to truncated proteins (*Arhgef10* fusion B). We also predict that subcellular localization of proteins translated from FTs may be changed, with the repeat-fusion creating or disrupting the targeting signal of an isoform (for instance, the N-terminal domain normally associated with the mitochondrial matrix localization of nuclear-encoded proteins). This may be a previously unappreciated way to increase the repertoire of isoforms of a gene/protein in the cell. However, it has yet to be determined that any of the FTs expressed in our data set gives rise to such proteins. Of note, a SINE-containing human brain-specific isoform of *Pgc1α*, which initiates transcription in a novel non-annotated upstream alternative promoter, has recently been reported and shown to be translated into protein [34].

Finally, using different statistical methods we identified FTs in which read counts were significantly different between saline- and cocaine-treated animals. However, qRT-PCR failed to validate some of the differential expression that was found to be statistically significant. In other cases, we found that qRT-PCR revealed differential expression that was not statistically significant by counting RNA-seq fusion reads. Some of this lack of reproducibility may be attributable to the natural biological variation between the two independent sets of animals used for the two different approaches. Another possibility is that the read counts for the fusion reads, which are only considered if they cross the exact fusion junction, may not adequately reflect the true level of expression of the entire FT. Until a better means of determining FT expression level is developed, alternative methods will be necessary to more accurately test differential expression of FTs in response to, for instance, an environmental exposure such as cocaine.

In conclusion, we developed a highly specific pipeline to detect FTs genome-wide using data resulting from widely available RNA-seq technology. While we based our analysis on the TopHat-Fusion platform, many fusion transcript detection software packages are available and could be the basis for similar analysis [35]. In fact, even aligners such as STAR (Spliced Transcripts Alignment to a Reference), which is known for its speed and its precision in detecting novel splicing junctions, could potentially be used [36]. However, none of these tools have been designed to detect repeat-exon fusion events like those described in this report. Thus, irrespective of the basic platform chosen and its inherent advantages and limitations, additional scripts similar to the ones developed by us for TopHat-Fusion in conjunction with application of strict filters and several rounds of optimization would be required for the accurate detection of events involving repeats. One of the advantages of our pipeline is that it supports both single- and paired-end reads, which is not the case for some of the other packages described [35]. The TopHat-Fusion approach has not only allowed the identification of hundreds of FTs in the NAc but will set the stage to better understand the biological significance of these forms of RNA, not only in the brain and in the context of cocaine, but also in other tissues. We envisage that similar mechanisms involving the expression of FTs are associated with the responses to many types of environmental exposures. This work thus opens new opportunities and challenges in addressing how the differential transcription of repeat-containing FTs contributes to development, cellular differentiation and disease states.

## Supporting Information

S1 Fig Schematic representation of analytical workflow using TopHat-Fusion. TopHat-Fusion was used to identify FTs. Reads were allowed to be mapped to one unique genomic locus on the reference genome to increase specificity of findings. The stringent criteria adopted to determine whether a transcript was expressed as a fusion are depicted in the figure.
(PDF)

S2 Fig FTs are expressed in the NAc. Reverse-transcription PCR was performed to determine expression of FTs in RNA samples from the NAc of animals exposed to saline or cocaine. *Arhgef10* fusion A (lane 1) and fusion B (lane 2) are depicted as representative of the fusion events validated. Gel has been cropped to remove irrelevant data; DNA marker was present in the same gel. Sequence of primers used for the PCR can be found in S2 Table.
(PDF)

S3 Fig Some genes expressing FTs are expressed in several tissues while others are expressed in a tissue-specific manner. Read counts were compared between FTs and non-fusion isoforms in different areas of the brain including the amigdala (AMY), hippocampus (HIP), NAc, prefrontal cortex (PFC) and the ventral tegmental area (VTA) as well as in different tissues. Black bars represent average counts from 4–6 independent samples of the fusion version of a transcript while white bars indicate the average for the non-fusion counterpart.
(PDF)

S4 Fig Regulation of fusion transcript expression differs from the non-fusion counterpart during development. Pattern of expression of fusion or non-fusion isoforms as a function of neocortex development. Bars represent average read counts ± SE from 2 samples (1 data set from males and other data set from female mice); thus statistical significance could not be tested. Data from 3 different layers were analyzed: IgL (infragranular layer), L4 (granular layer) and SgL (supragranular layer). P4–14 represent post-natal days 4 to 14.
(PDF)

S5 Fig Architecture and types of FTs detected in the NAc. Representation of the fusion events predicted in 24 additional randomly selected genes out of the 813 events detected. The canonical start codon (ATG) is shown based on gene annotation; directionally of transcription is indicated with red arrow above and below the panels based on UCSC gene annotation. In all cases, top panel shows full view of gene structure. TSS indicates the transcription start site. Middle and/or lower panels show enlarged view of different predicted fusions, including the splicing recognition sites (GT-AG). Orange or green brackets indicate regions involved in the fusion (repeat/exon or vice-versa). Red boxes indicate the region from which RNA-seq reads support the fusion events; when more than one fusion was identified on the same gene (isoform) green boxes identify the reads supporting the additional event. The number of reads supporting the fusion event is also depicted.
(PDF)

S6 Fig Epigenetic marks associated with promoters are identified on the SSR present in the newly identified *Atp2b1* fusion. ChIP-seq was performed using different histone marks and RNA polymerase II (for more details see reference 19). The different histone marks analyzed are shown on the first 3 tracks; Pol II peak is also included (green) as are the RNA-seq counts (blue). The position of the annotated SSR used as promoter in this fusion is shown below in light gray based on RepeatMasker.
(PDF)

S7 Fig Conservation of the SSR present in the *Atp2b1* fusion. RepeatMasker track indicate in grey the SSR involved in this fusion. Below the nucleotide sequence of the repeat in various organisms is shown. The chromosomal coordinates for the SSR is shown above the figure.
(PDF)

S8 Fig Conservation of the SINE and SSR present in the *Pgc1α* fusion in different species. RepeatMasker track indicate in grey the SINE (upper panel) and the SSR (lower panel) involved in this fusion. The nucleotide sequence of the repeat in various organisms is shown below the RepeatMasker track; the genomic coordinates of each individual repeat are shown above each panel on the figure.
(PDF)

S1 Table Full list of the 438 genes that express 813 FTs as identified by the TopHat-Fusion analysis. Gene name, fusion type, chromosomal coordinates, direction of the fusion and read count in saline- or cocaine-treated samples are shown. The type of repeat, the family and class of the RE as well as the strand of the elements involved in the fusion are also depicted. S1–3 reflect each of the cocaine samples (5 animals in each sample) and S4–6 refer to saline-treated controls (5 animals/sample). Statistical analysis was performed based on raw read counts as well as fold-change using Wilcoxon test. In bold are the p-values that are statistically significant with p≤0.05.
(XLSX)

S2 Table Primer sequences for fusion validation and expression comparison. Forward (F) and reverse (R) primers were designed to amplify the RE and exon present in each FT. In some cases the same exon was amplified with a primer from an adjacent exon in order to compare the level of the FT with that of the non-fusion isoform (control locus, con).
(XLSX)

S3 Table Expected versus observed frequency of repeats in each fusion transcript. The list of 813 FTs (from S1 Table) was used to calculate the observed frequency of repeats per RE class relative to the expected frequency considering the genomic annotation of the repeats. Depicted on the table are the RE class, the number (#) of events observed involved REs from each class, the percentage (%) of the event relative to the total number as well as the expected values considering repeats that are annotated in an intron or intergenic region. Data depicted is derived from “plus” strand of the genome only.
(XLSX)

S4 Table Full list of candidate genes to express FTs as per the hard clipping analysis. Only gene name is shown. In bold are the genes also identified by the TopHat-Fusion pipeline as FT-expressing genes.
(XLSX)
