# Supplementary figures and images for: A Novel Analytical Strategy to Identify Fusion Transcripts between Repetitive Elements and Protein Coding-Exons Using RNA-Seq
Source: PLoS One. 2016 Jul 14;11(7):e0159028. doi: 10.1371/journal.pone.0159028 (PMC4945064; doi:10.1371/journal.pone.0159028)

S1 Figure

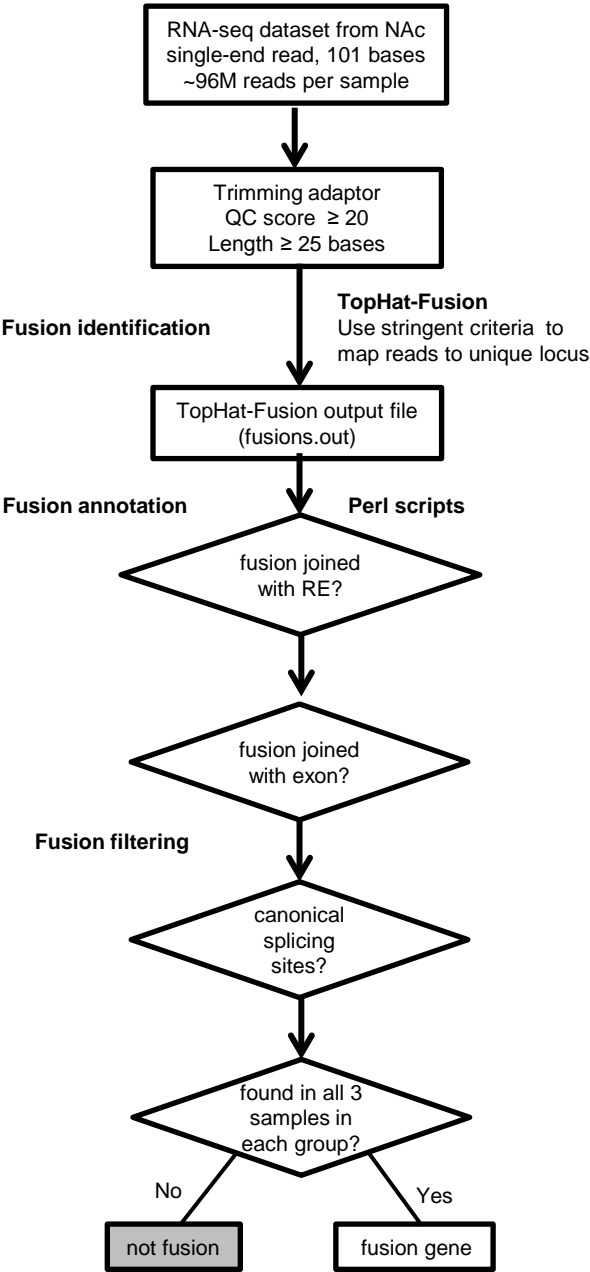

Supplement: S1 Fig — TopHat-Fusion was used to identify FTs. Reads were allowed to be mapped to one unique genomic locus on the reference genome to increase specificity of findings. The stringent criteria adopted to determine whether a transcript was expressed as a fusion are depicted in the figure. (PDF) [file pone.0159028.s001.pdf]

S2 Figure

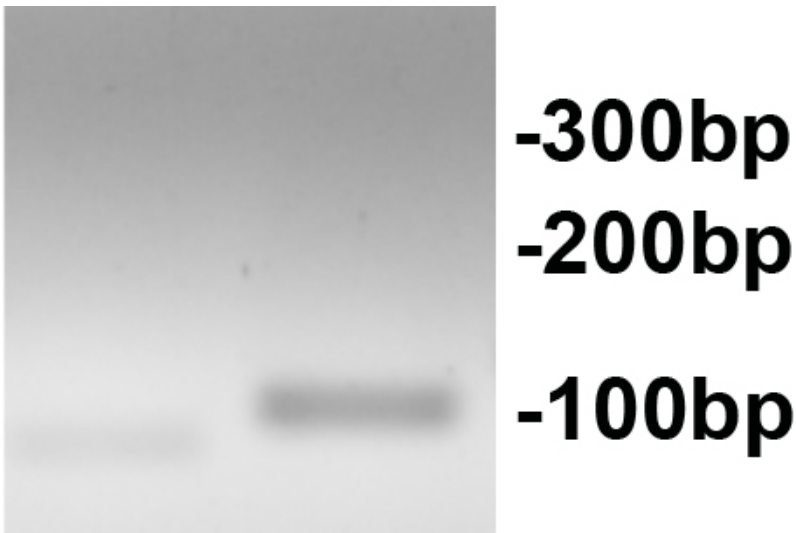

Supplement: S2 Fig — Reverse-transcription PCR was performed to determine expression of FTs in RNA samples from the NAc of animals exposed to saline or cocaine. Arhgef10 fusion A (lane 1) and fusion B (lane 2) are depicted as representative of the fusion events validated. Gel has been cropped to remove irrelevant data; DNA marker was present in the same gel. Sequence of primers used for the PCR can be found in S2 Table. (PDF) [file pone.0159028.s002.pdf]

S3 Figure

A

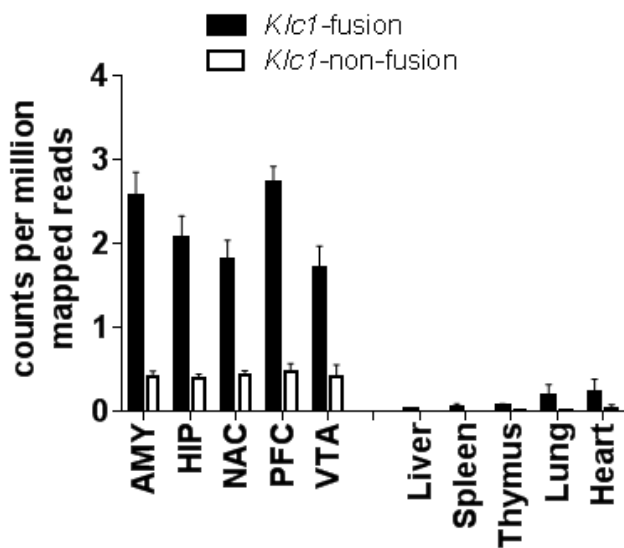

B

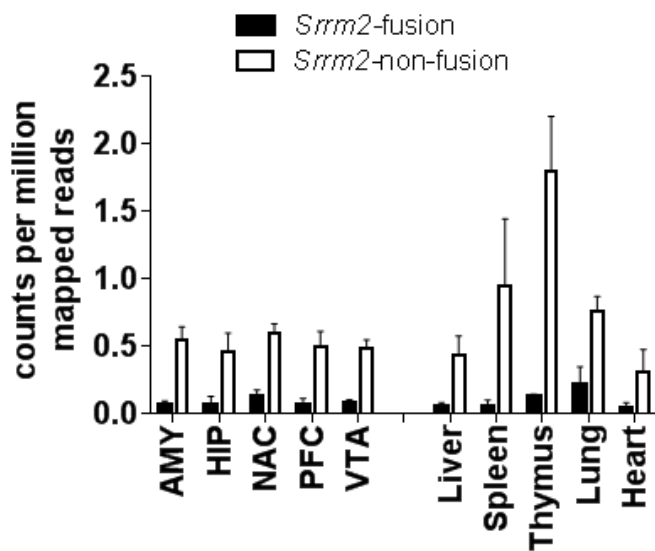

C

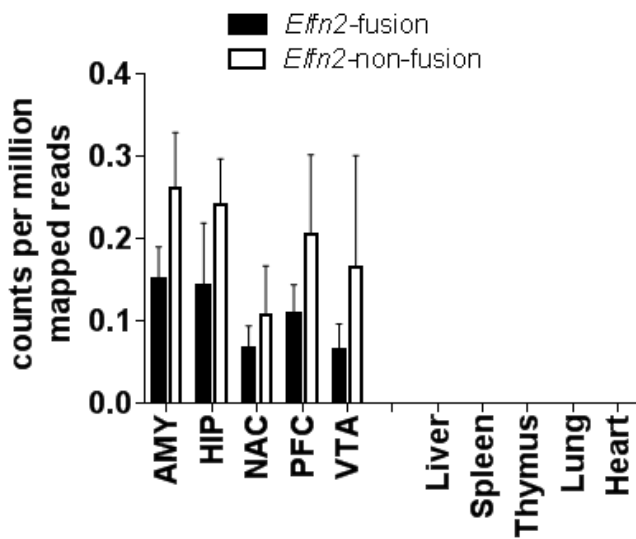

S3 Figure  
D

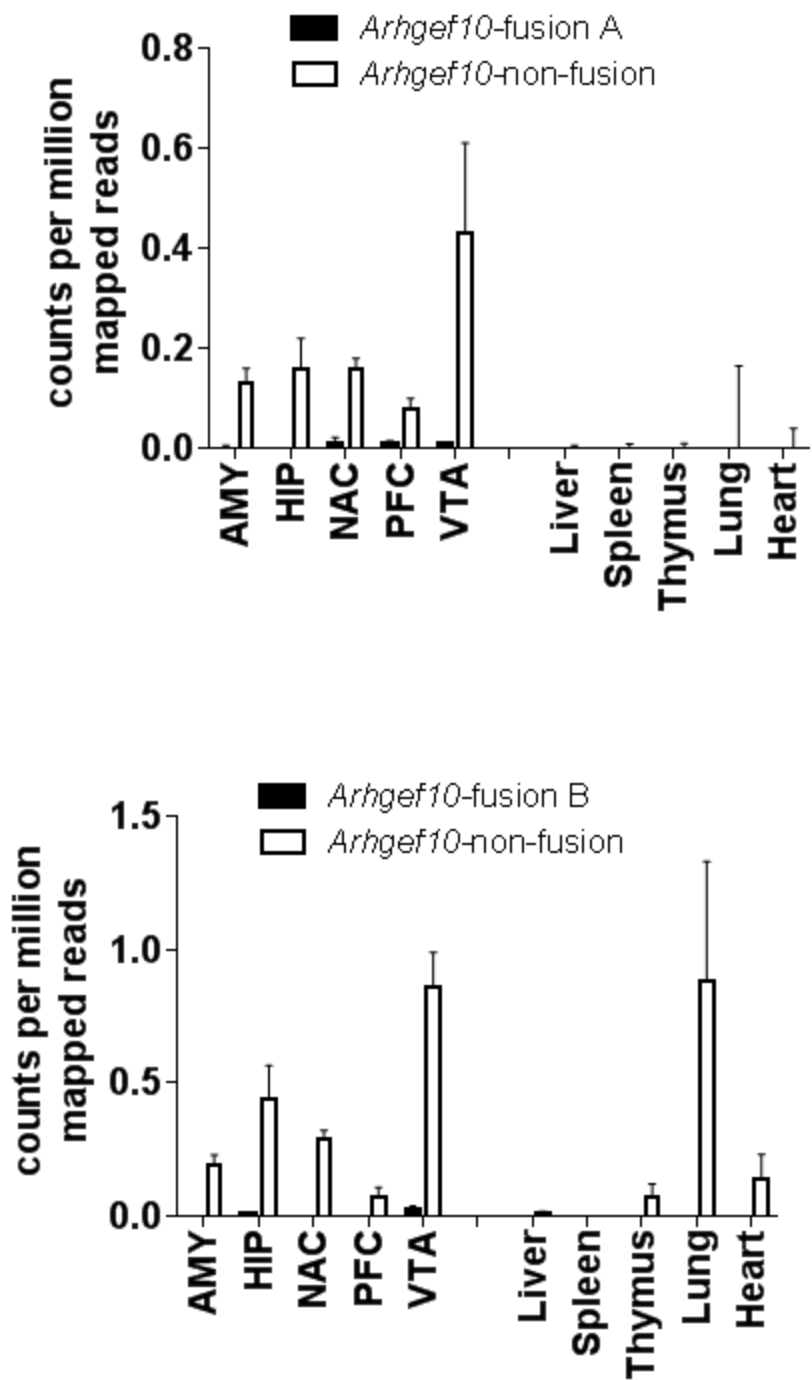

Supplement: S3 Fig — Read counts were compared between FTs and non-fusion isoforms in different areas of the brain including the amigdala (AMY), hippocampus (HIP), NAc, prefrontal cortex (PFC) and the ventral tegmental area (VTA) as well as in different tissues. Black bars represent average counts from 4–6 independent samples of the fusion version of a transcript while white bars indicate the average for the non-fusion counterpart. (PDF) [file pone.0159028.s003.pdf]

S4 Figure

A

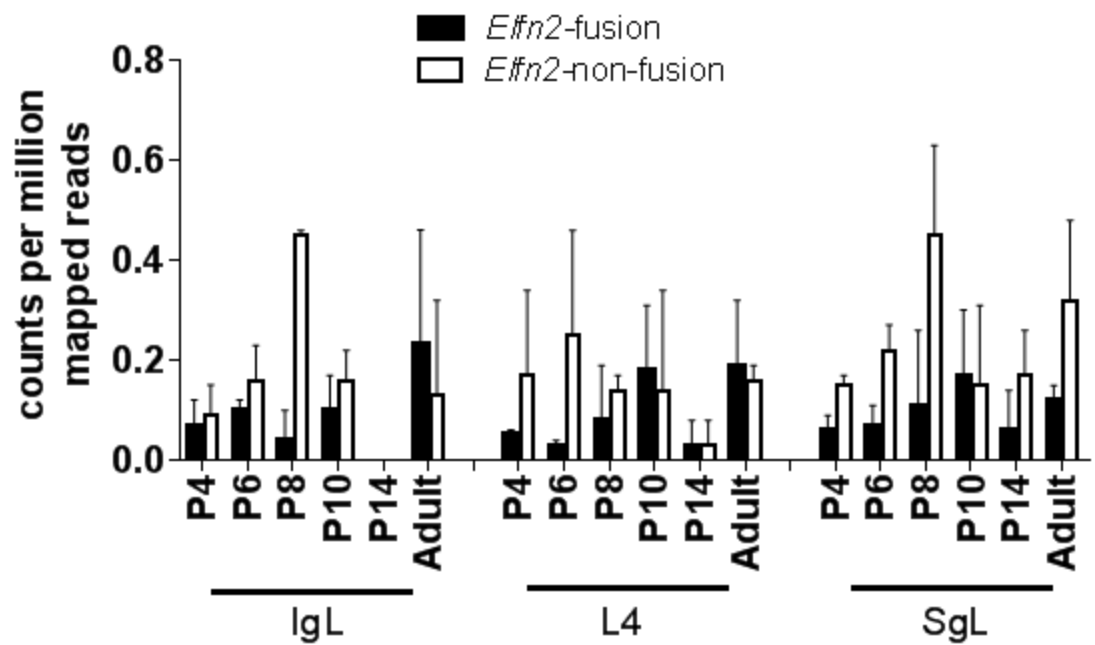

B

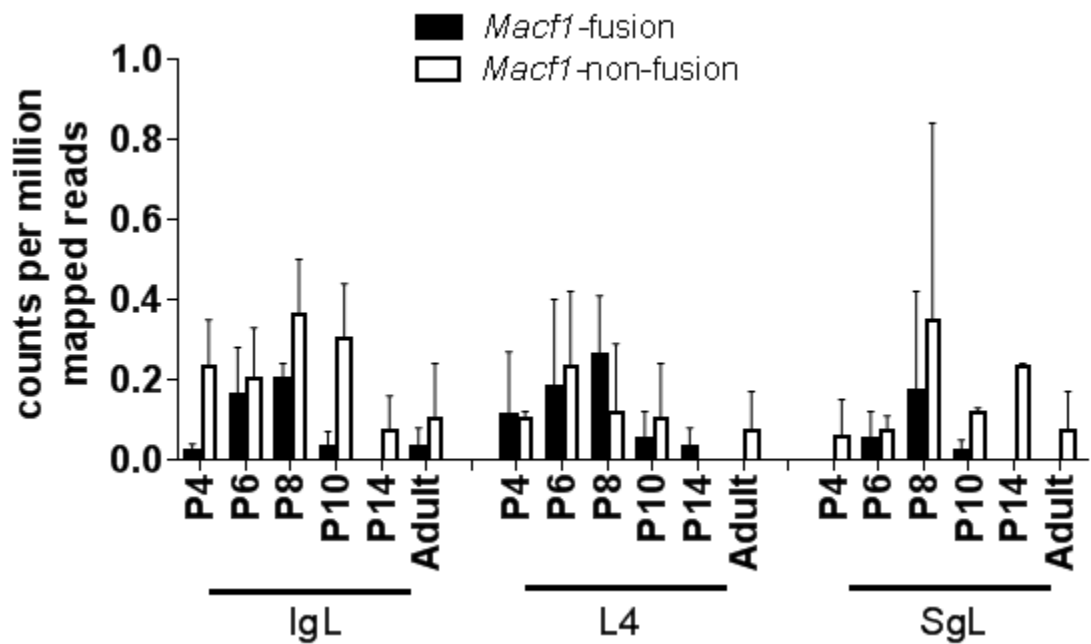

S4 Figure  
c

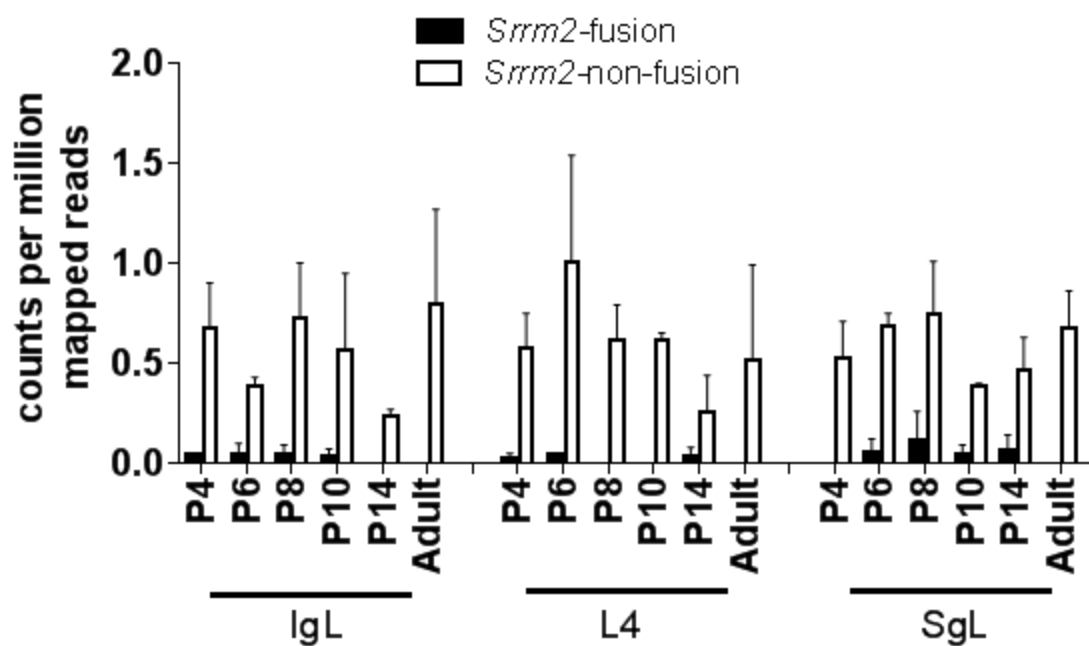

D

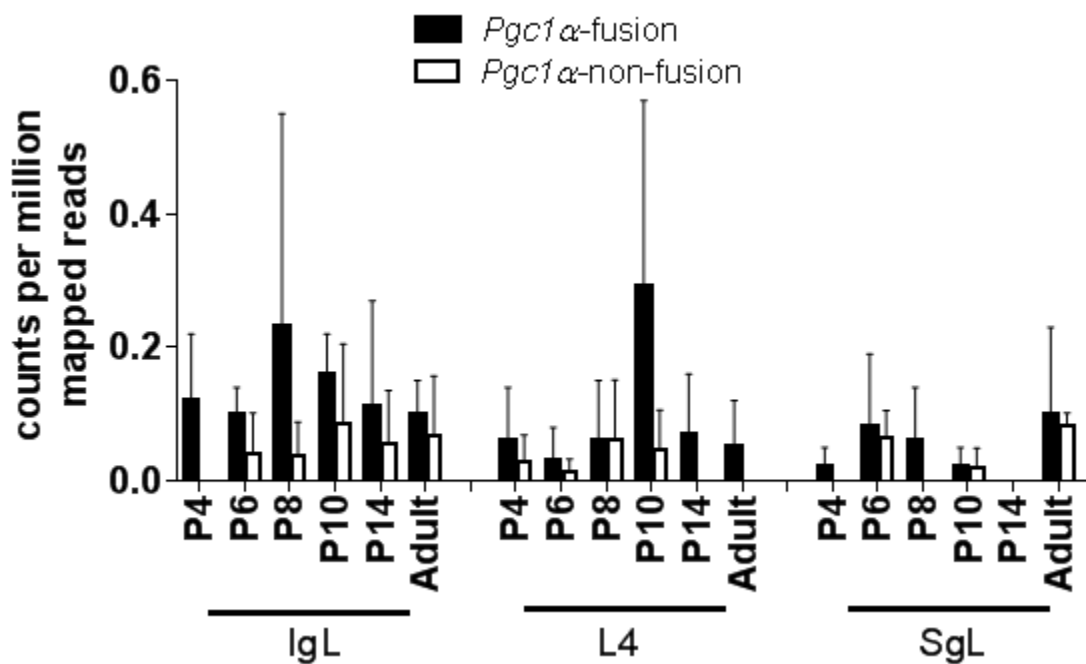

S4 Figure  
E

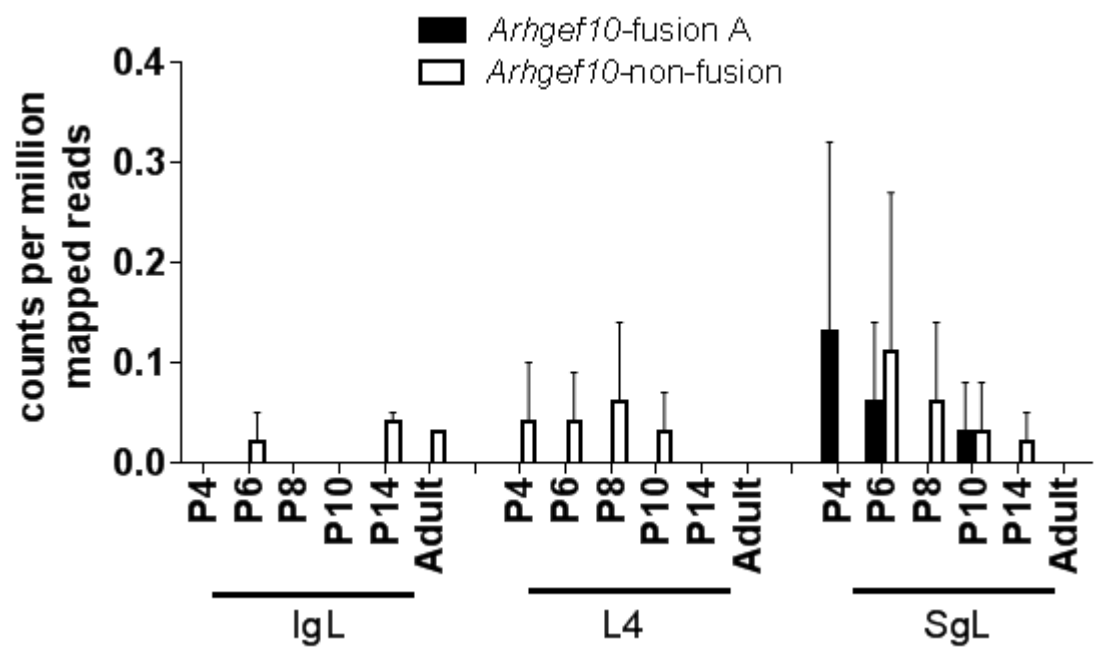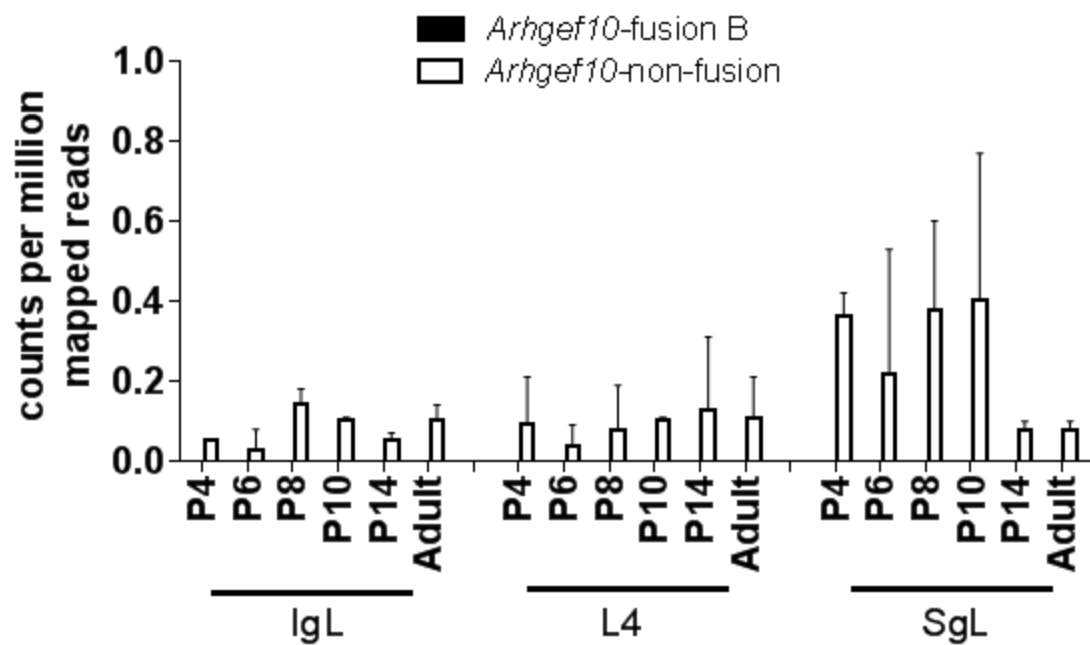

Supplement: S4 Fig — Pattern of expression of fusion or non-fusion isoforms as a function of neocortex development. Bars represent average read counts ± SE from 2 samples (1 data set from males and other data set from female mice); thus statistical significance could not be tested. Data from 3 different layers were analyzed: IgL (infragranular layer), L4 (granular layer) and SgL (supragranular layer). P4–14 represent post-natal days 4 to 14. (PDF) [file pone.0159028.s004.pdf]

S6 Figure

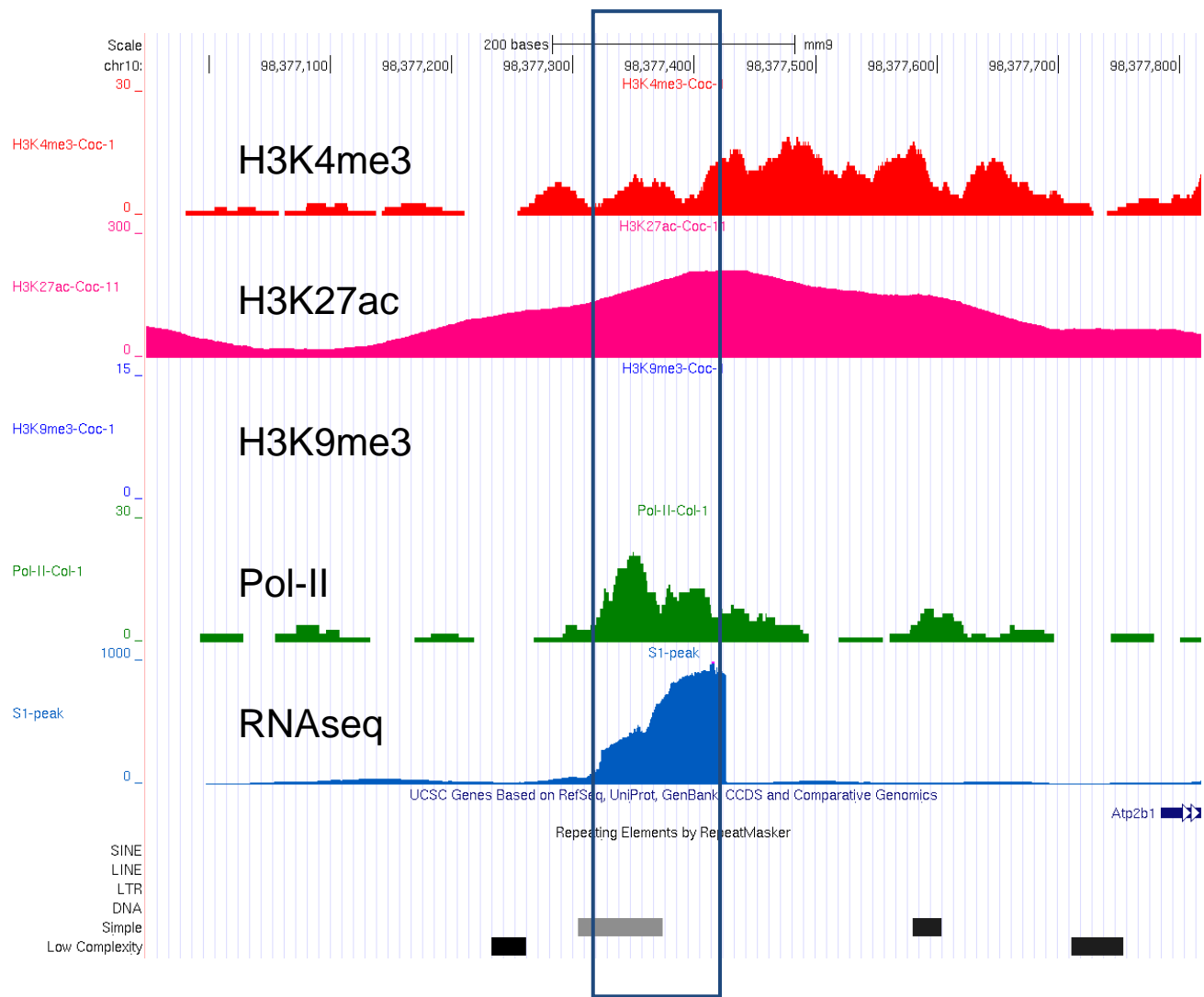

Supplement: S6 Fig — ChIP-seq was performed using different histone marks and RNA polymerase II (for more details see reference 19). The different histone marks analyzed are shown on the first 3 tracks; Pol II peak is also included (green) as are the RNA-seq counts (blue). The position of the annotated SSR used as promoter in this fusion is shown below in light gray based on RepeatMasker. (PDF) [file pone.0159028.s006.pdf]

# S7 Figure

SSR coordinates chr10: 98,377,341 - 98,377,427

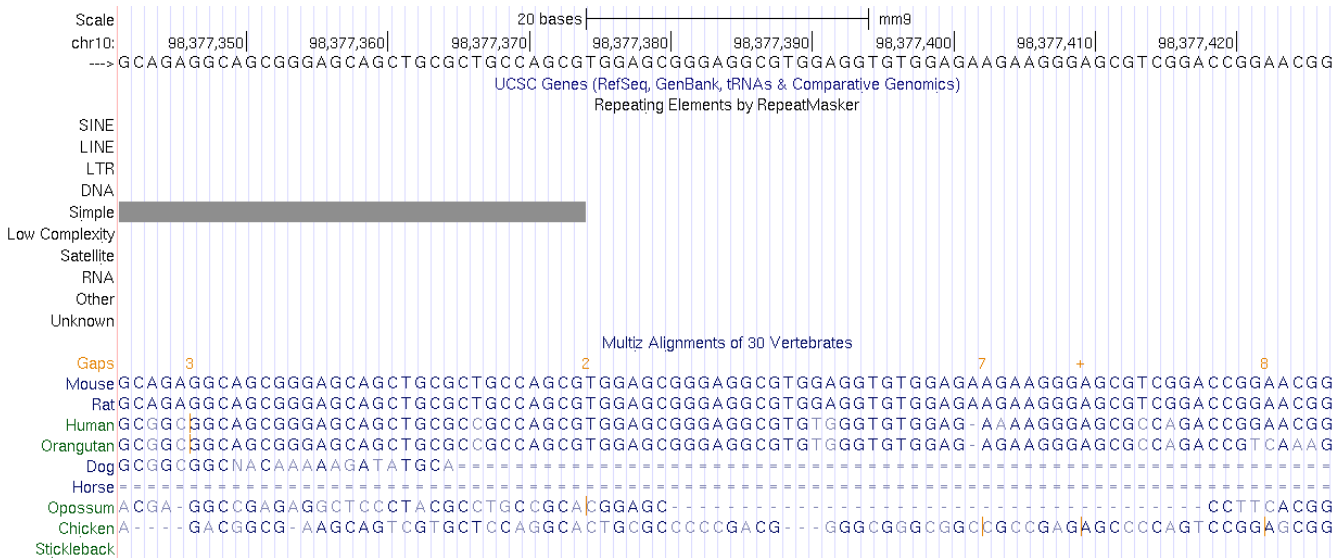

Supplement: S7 Fig — RepeatMasker track indicate in grey the SSR involved in this fusion. Below the nucleotide sequence of the repeat in various organisms is shown. The chromosomal coordinates for the SSR is shown above the figure. (PDF) [file pone.0159028.s007.pdf]

SINE coordinates chr5: 52,303,944 - 52,304,006

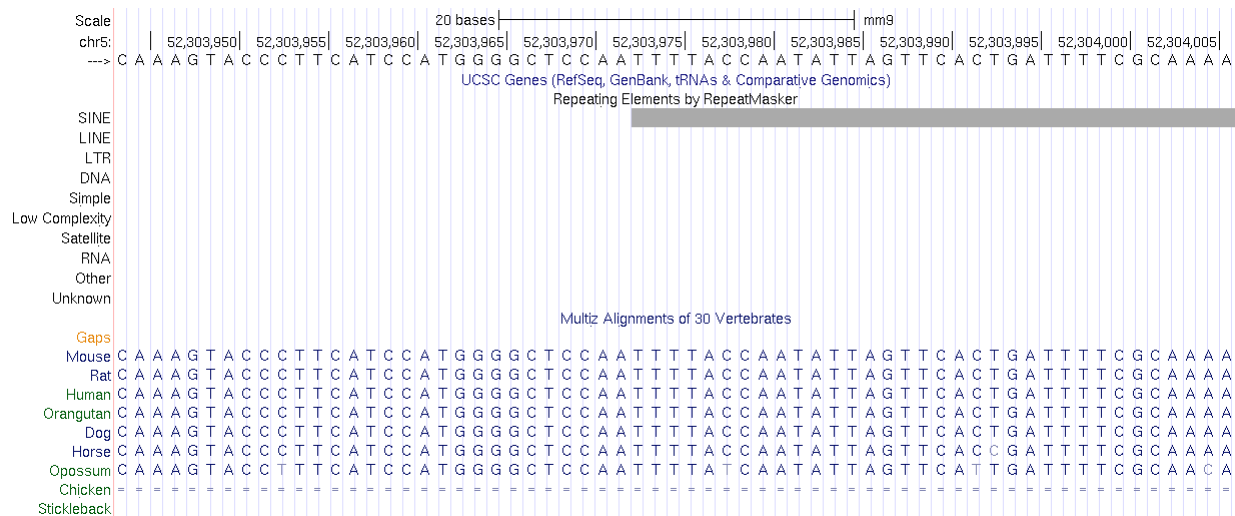

## SSR coordinates Region chr5: 52,506,600 - 52,507,200

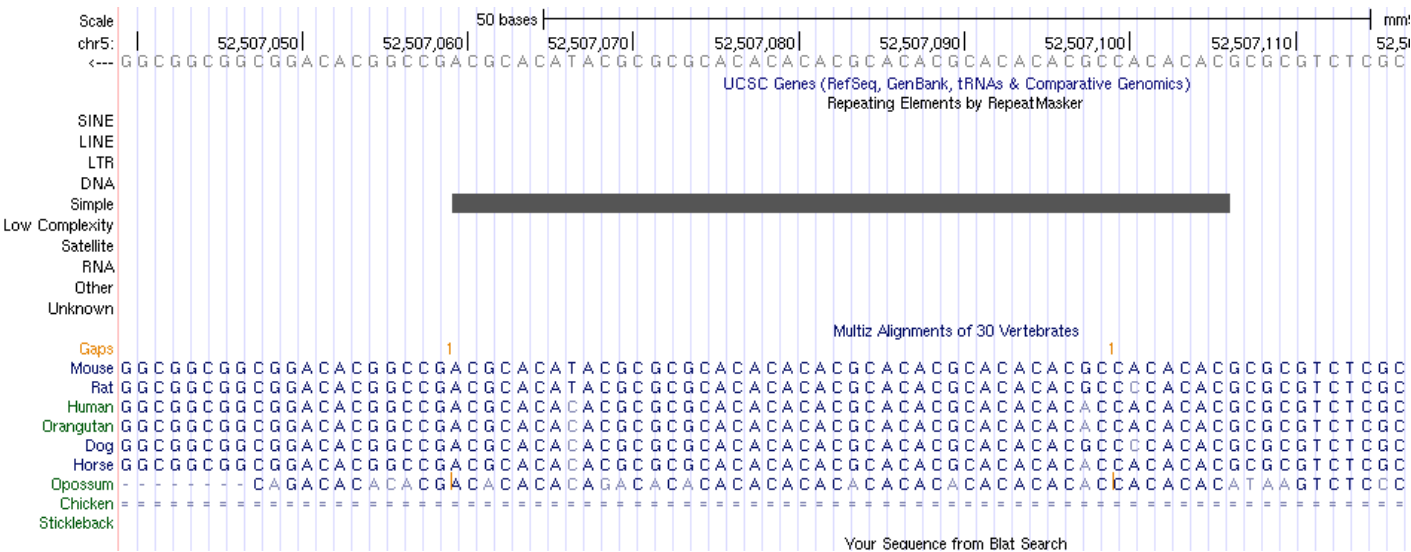

Supplement: S8 Fig — RepeatMasker track indicate in grey the SINE (upper panel) and the SSR (lower panel) involved in this fusion. The nucleotide sequence of the repeat in various organisms is shown below the RepeatMasker track; the genomic coordinates of each individual repeat are shown above each panel on the figure. (PDF) [file pone.0159028.s008.pdf]
