# Supplementary material for: A Novel Analytical Strategy to Identify Fusion Transcripts between Repetitive Elements and Protein Coding-Exons Using RNA-Seq
Source: PLoS One. 2016 Jul 14;11(7):e0159028. doi: 10.1371/journal.pone.0159028 (PMC4945064; doi:10.1371/journal.pone.0159028)

S5 Figure

4930402H24Rik fusions with LINE

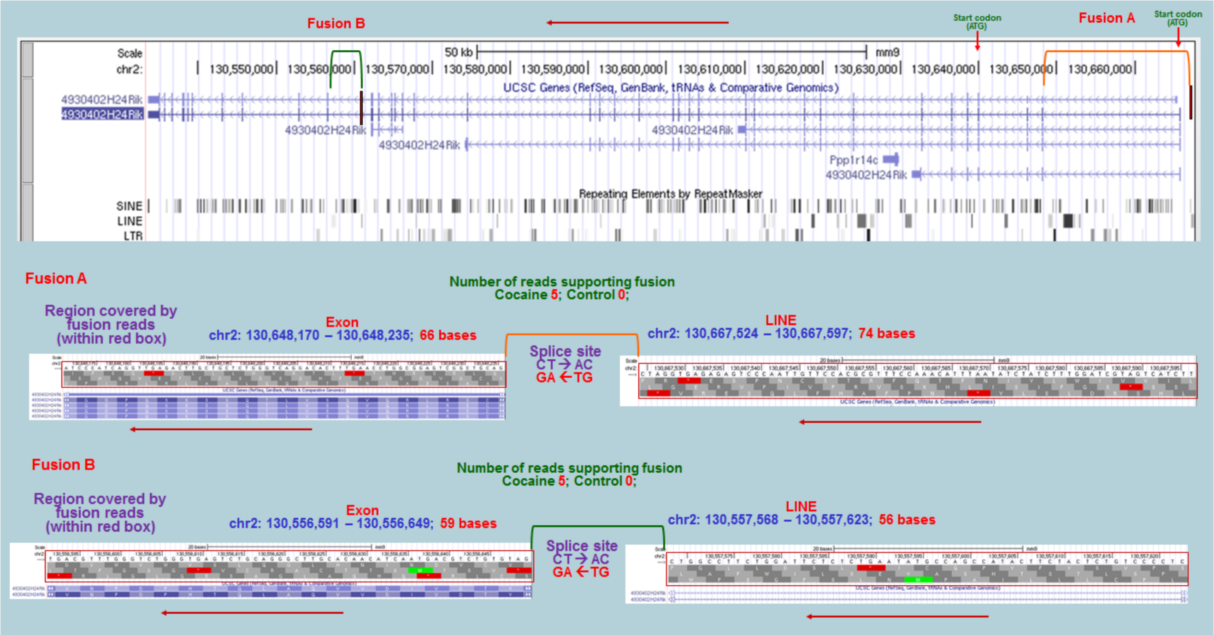

Atp5I fusion with LINE

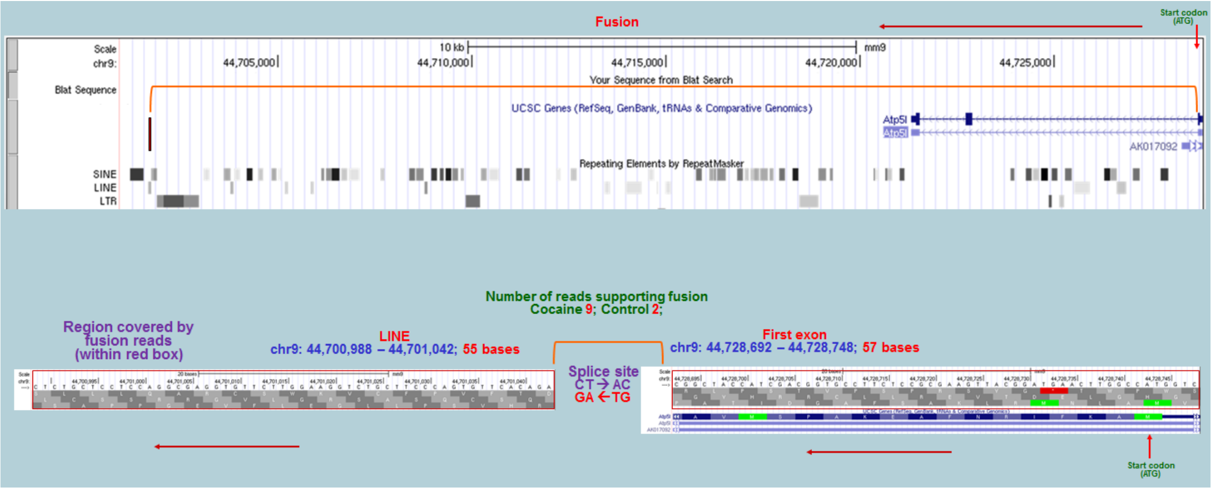

BC020535 fusions with SINE

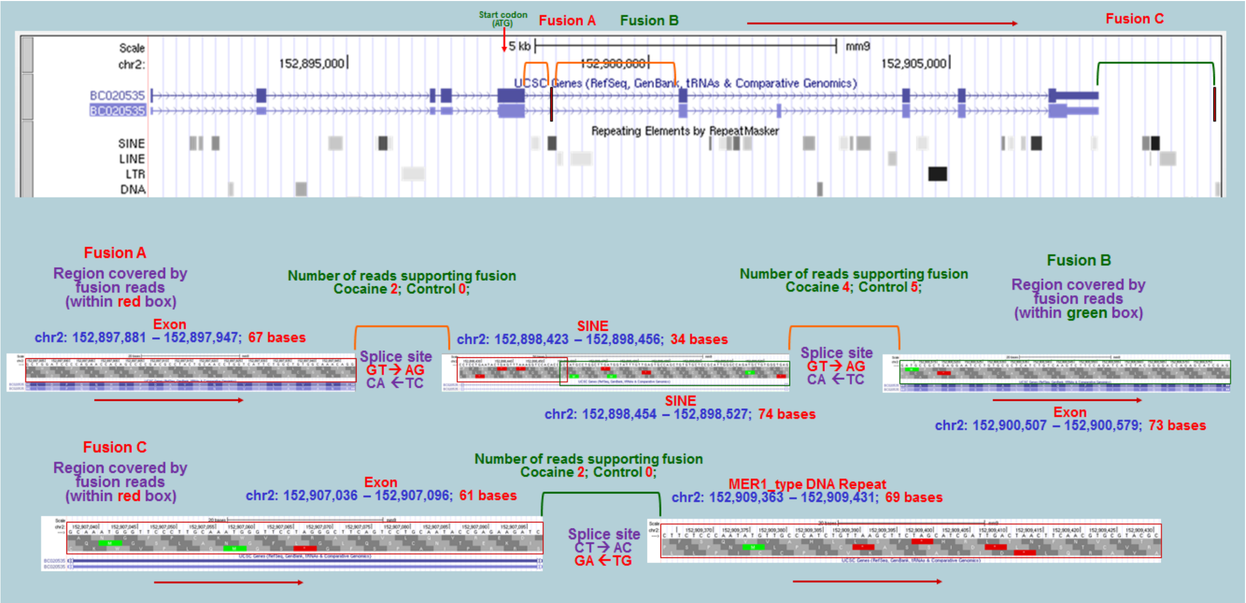

Ccdc126 fusions with LINE

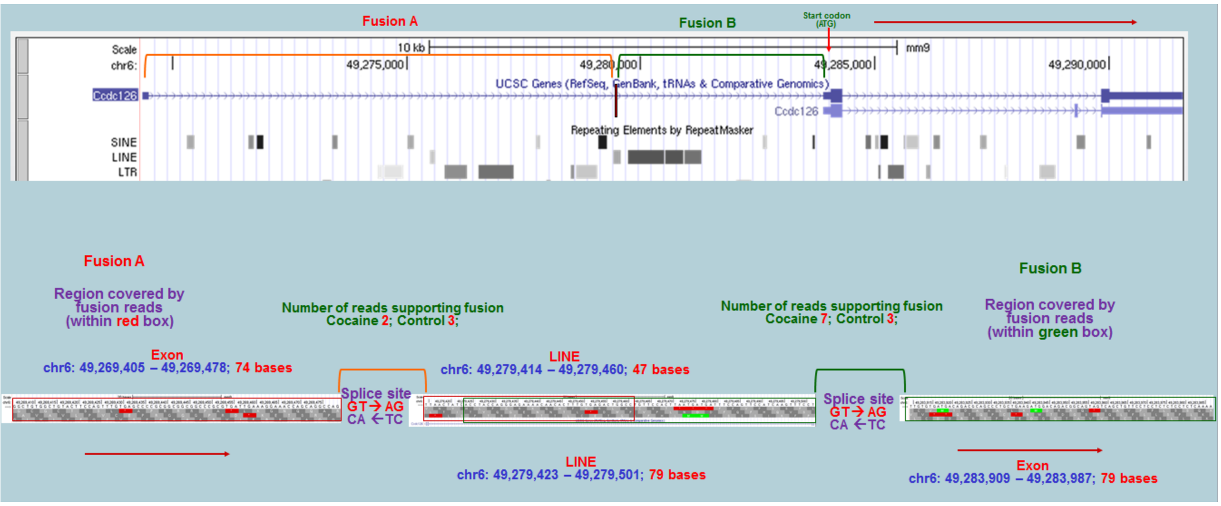

Cdadc1 fusion with SINE

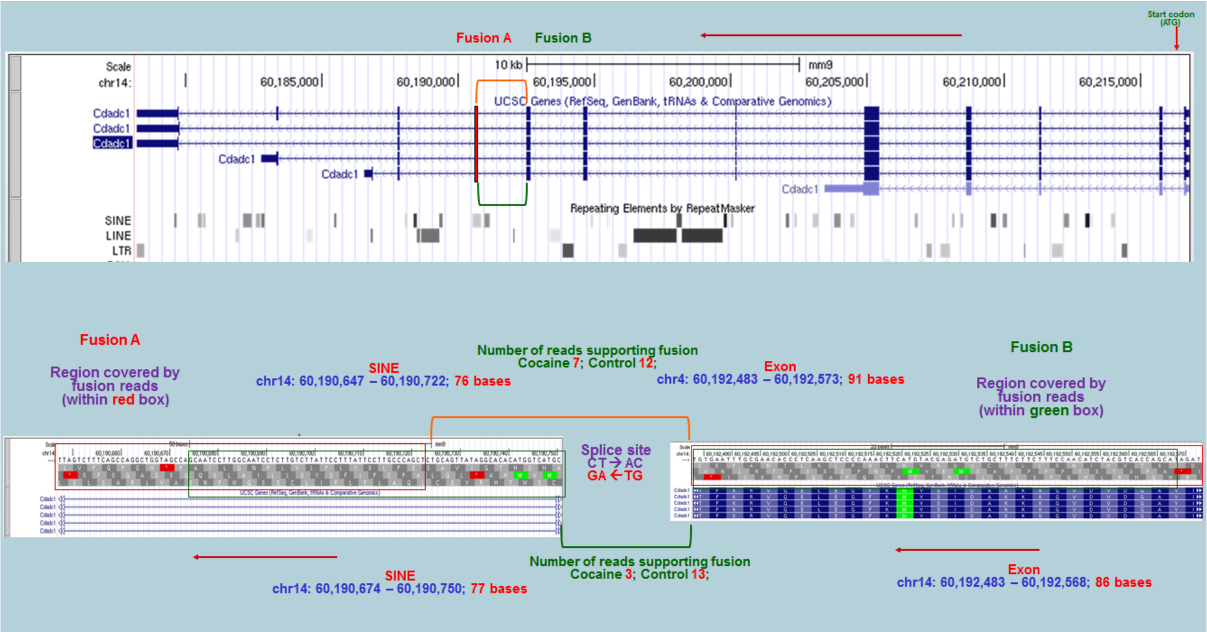

Celf2 fusion with Simple repeat

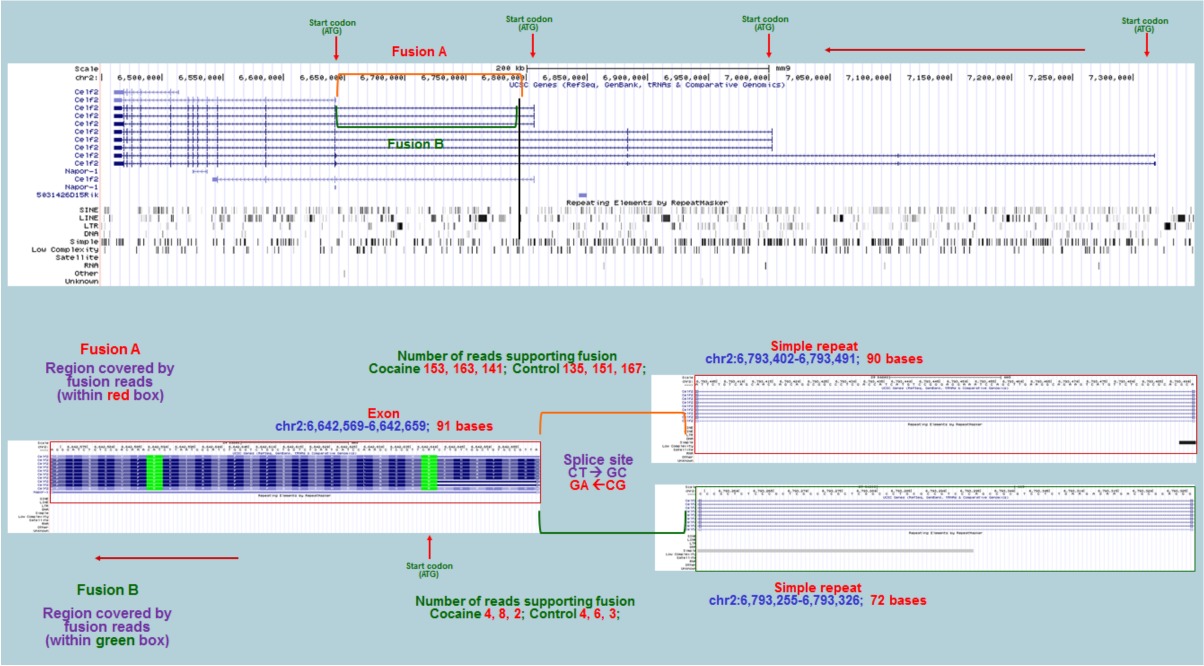

Ddr2 fusions with SINE

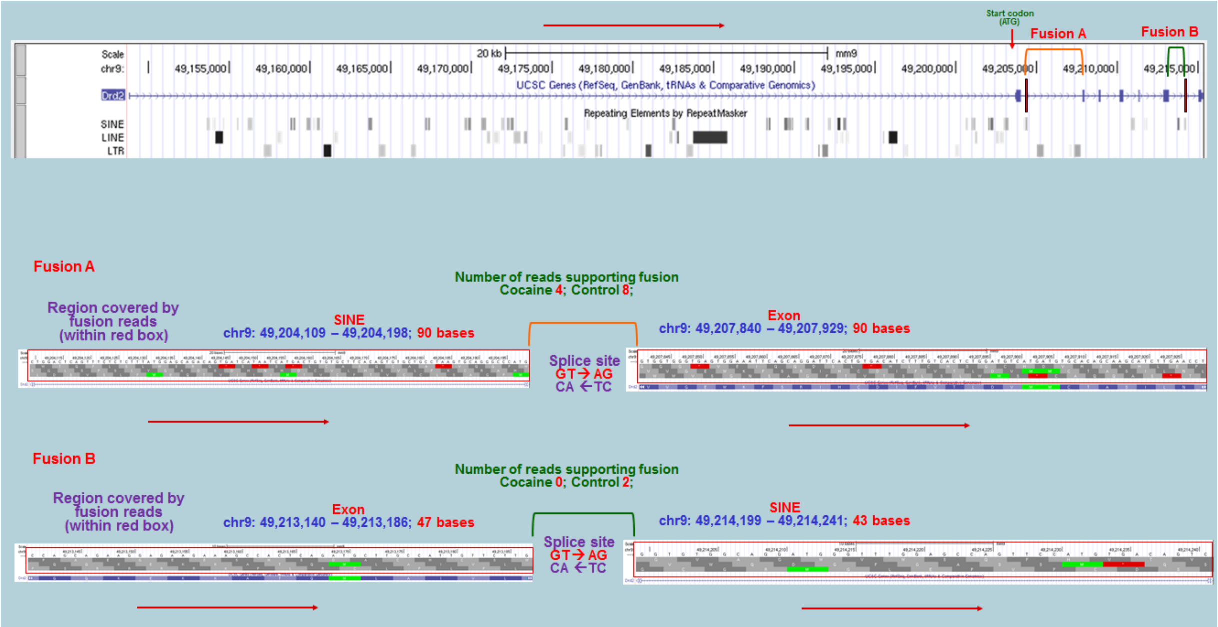

Dhx34 fusion with SINE

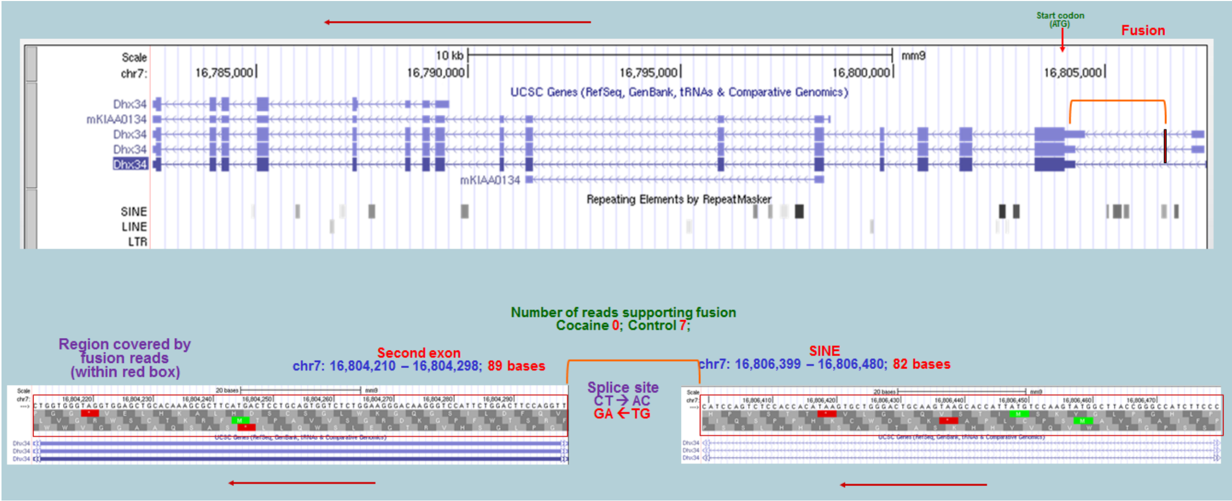

Elfn2 fusion with LINE

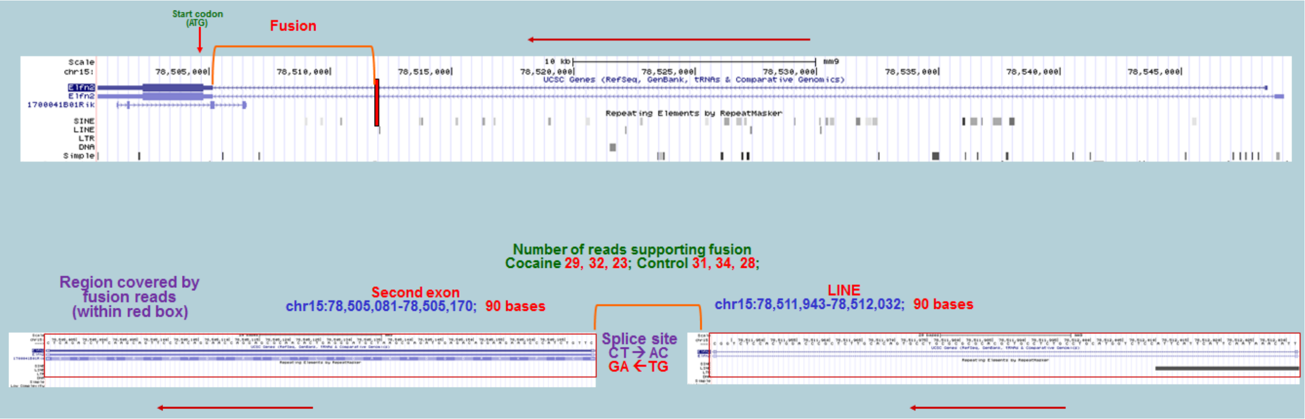

Fbf1 fusions with SINE

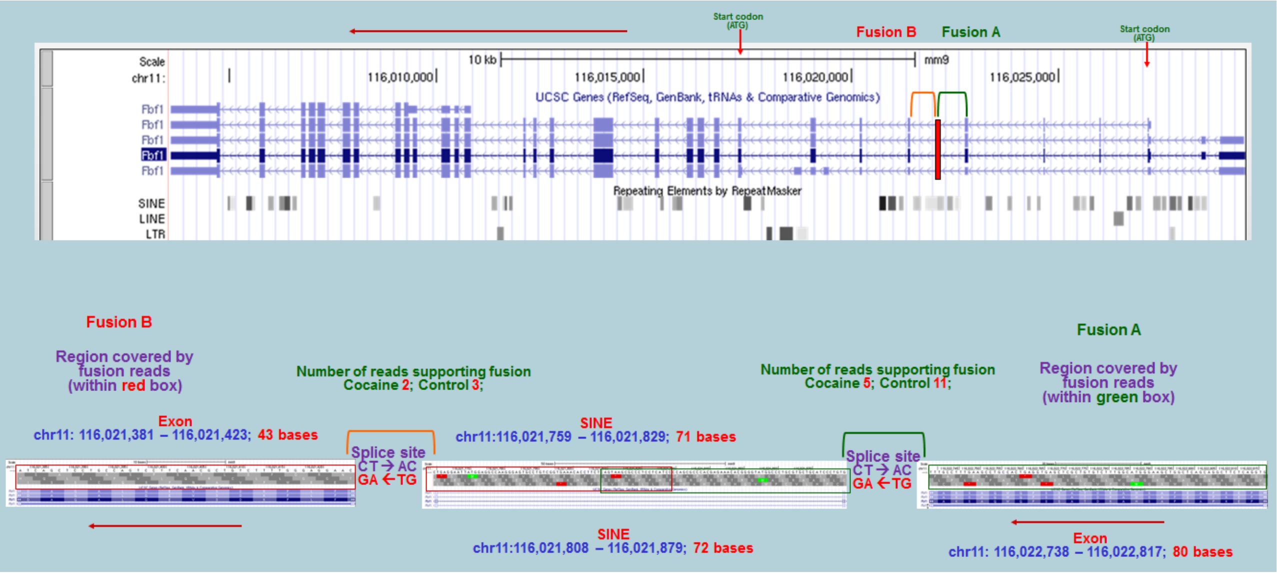

Foxk2 fusion with SINE

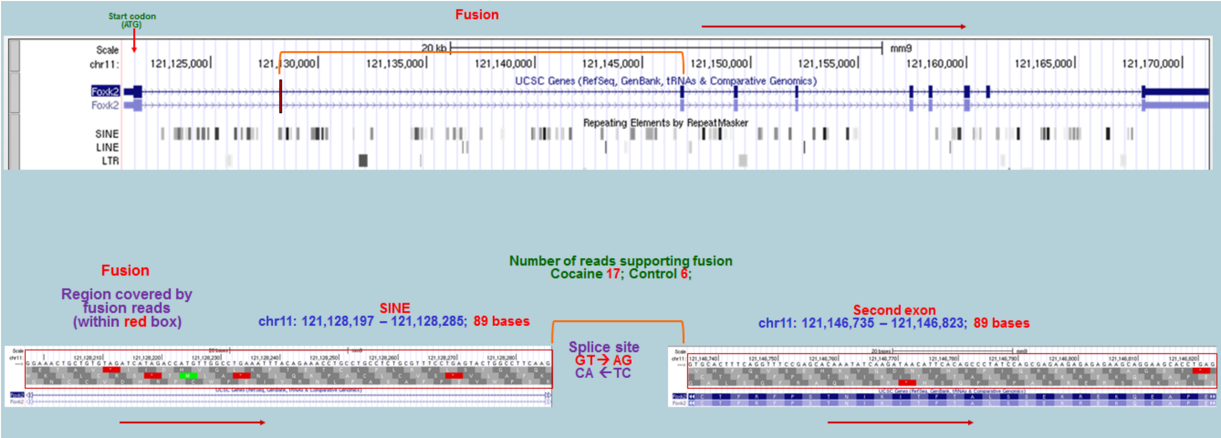

Fyco1 fusions with SINE

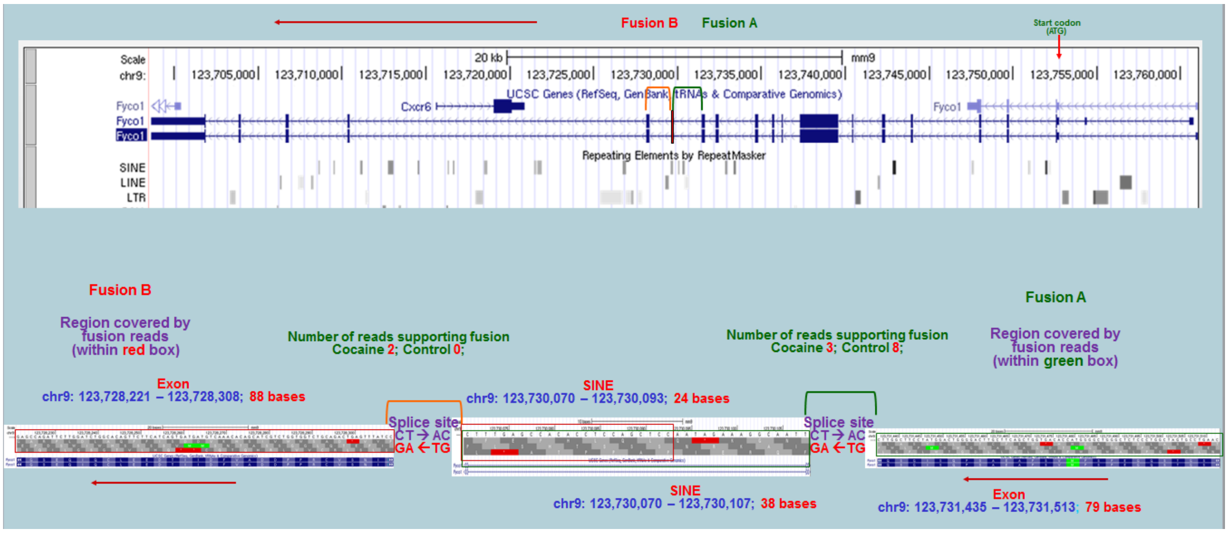

Gm996 fusion with

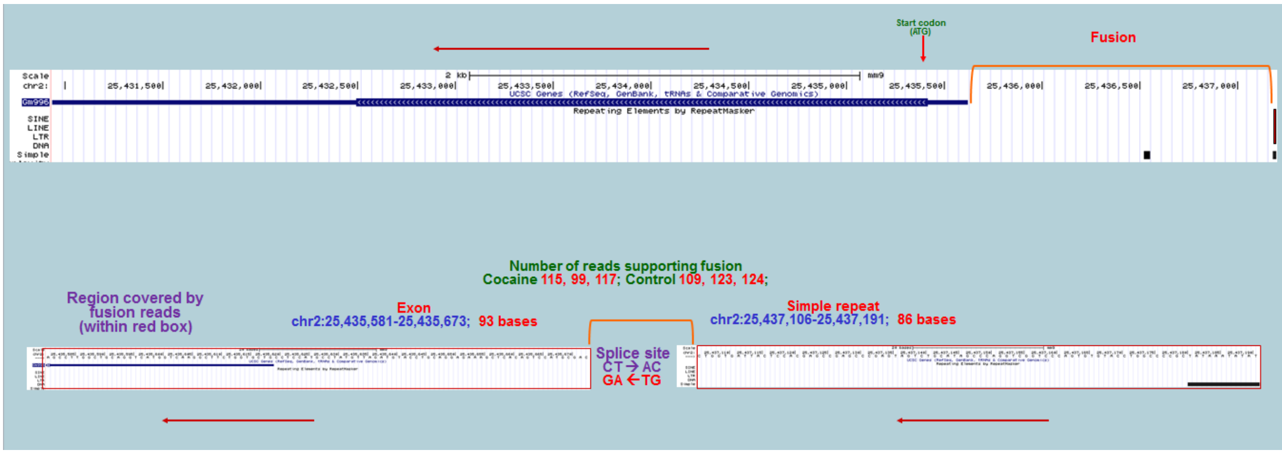

Hdac7 fusions with low-complexity repeat

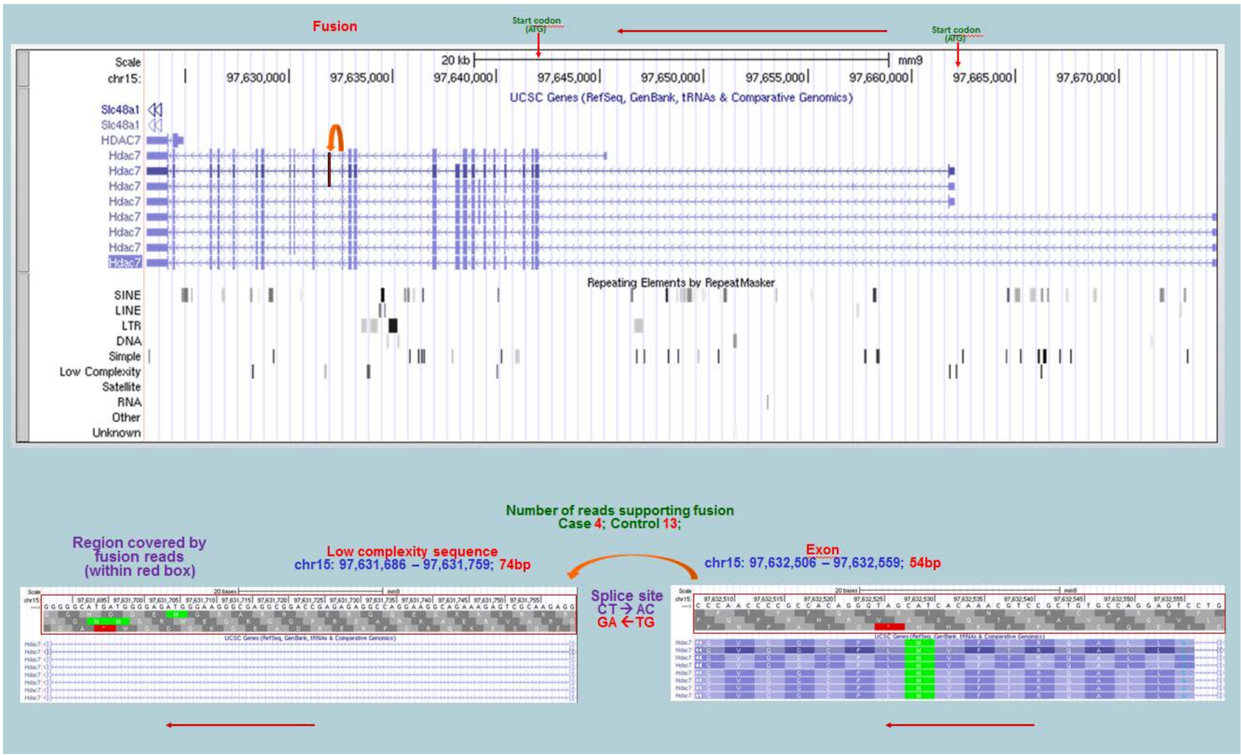

Idh2 fusions with SINE

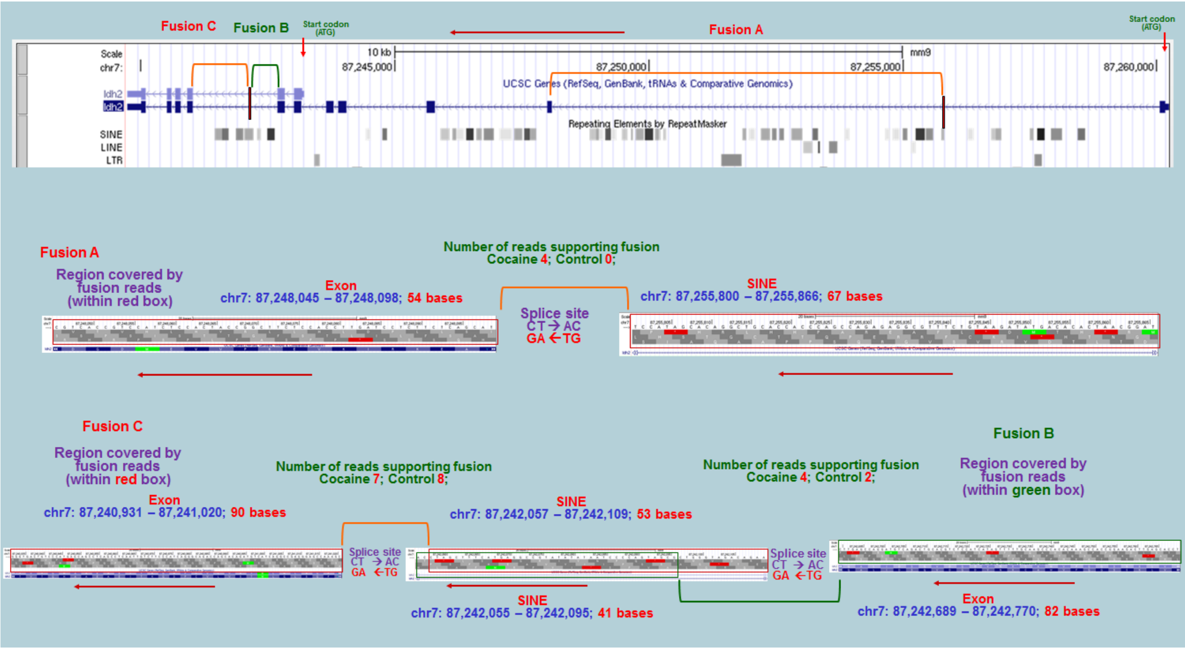

Kcnab2 fusion with LTR

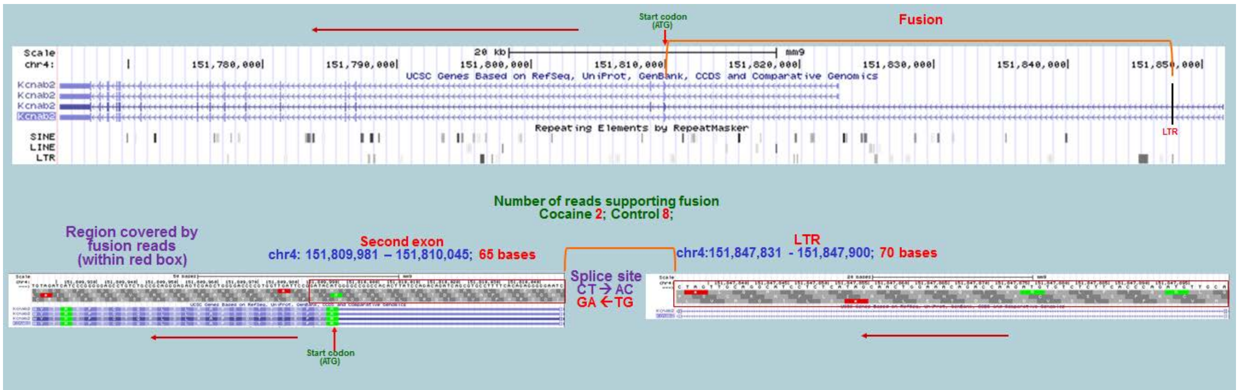

Kcnmb4 fusion with LTR

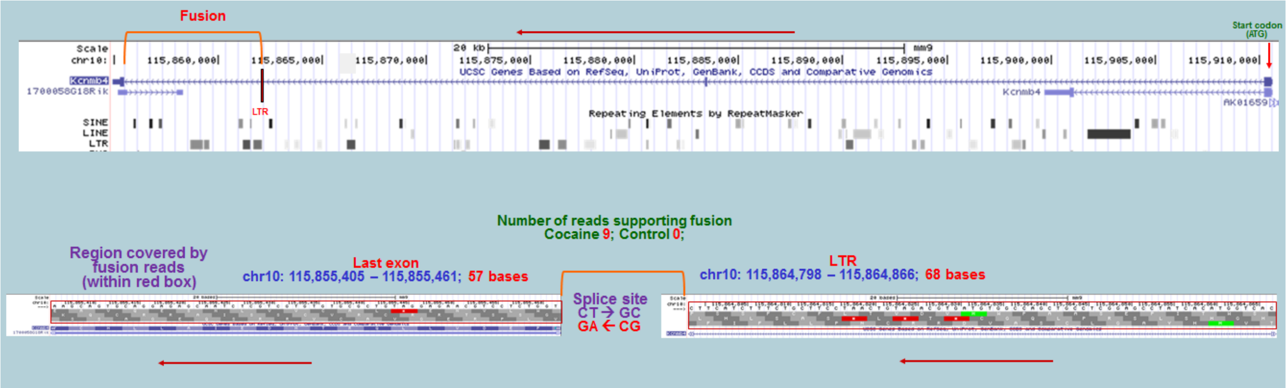

Klc1 fusion with SINE

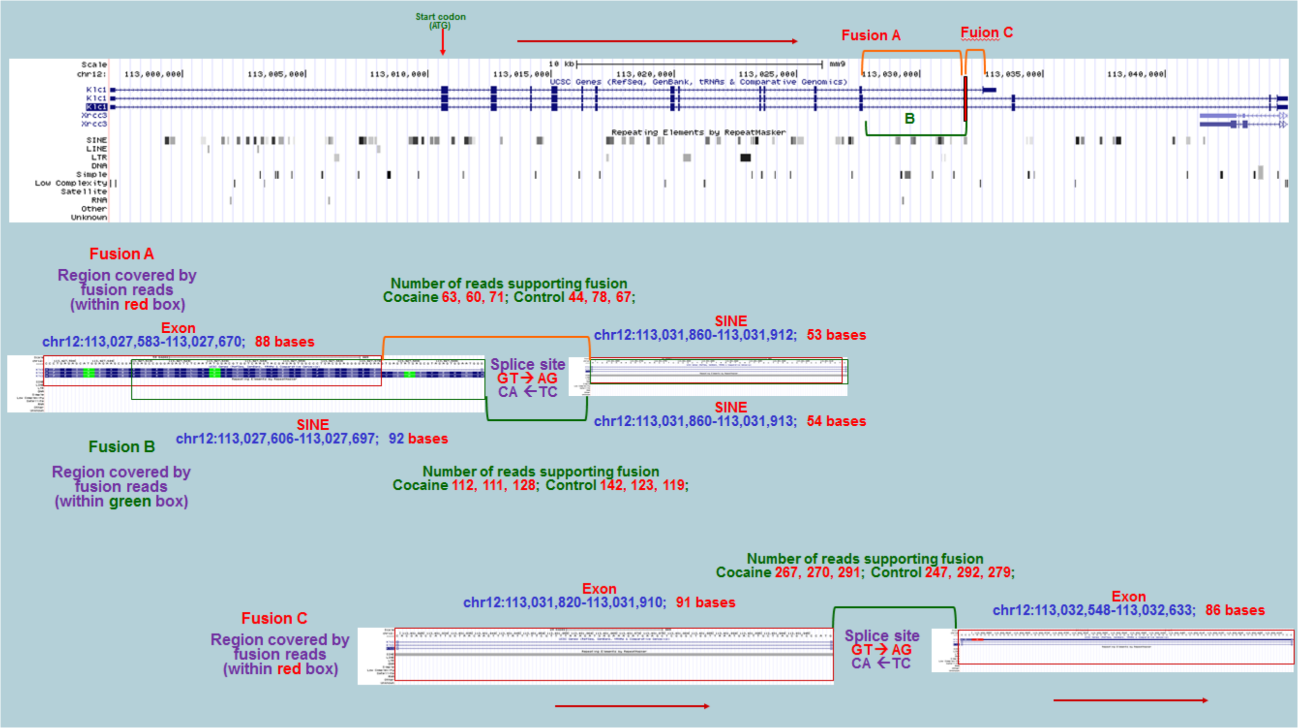

Macf1 fusion with low-complexity repeat

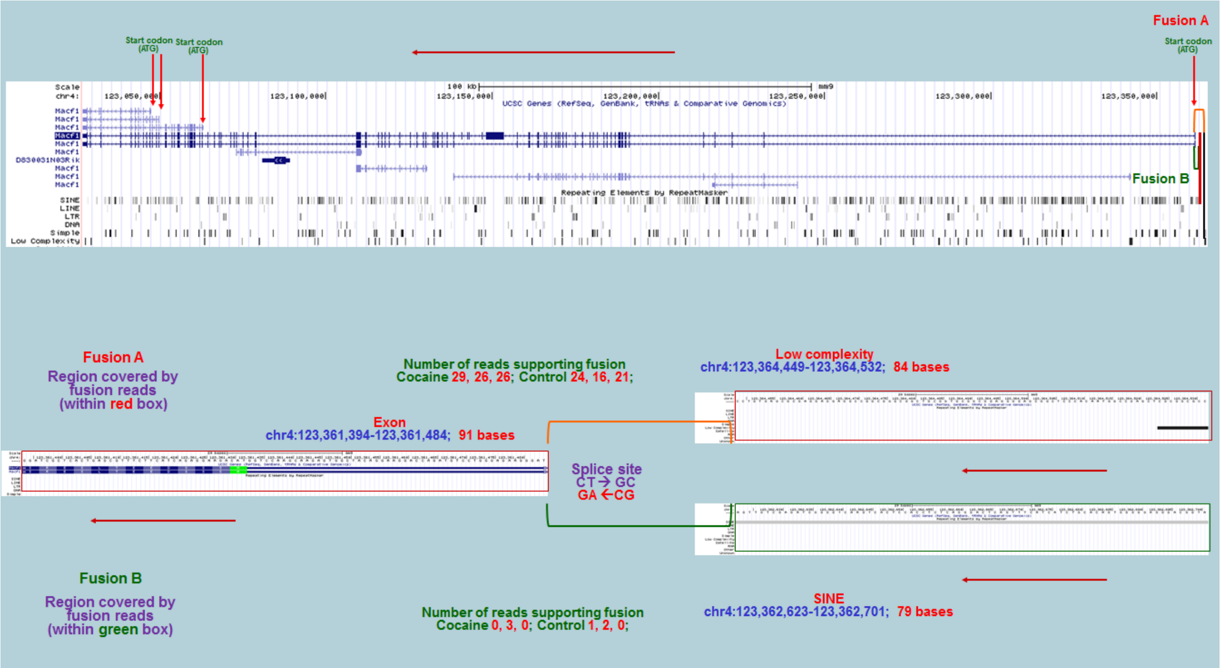

Ndufs8 fusion with SINE

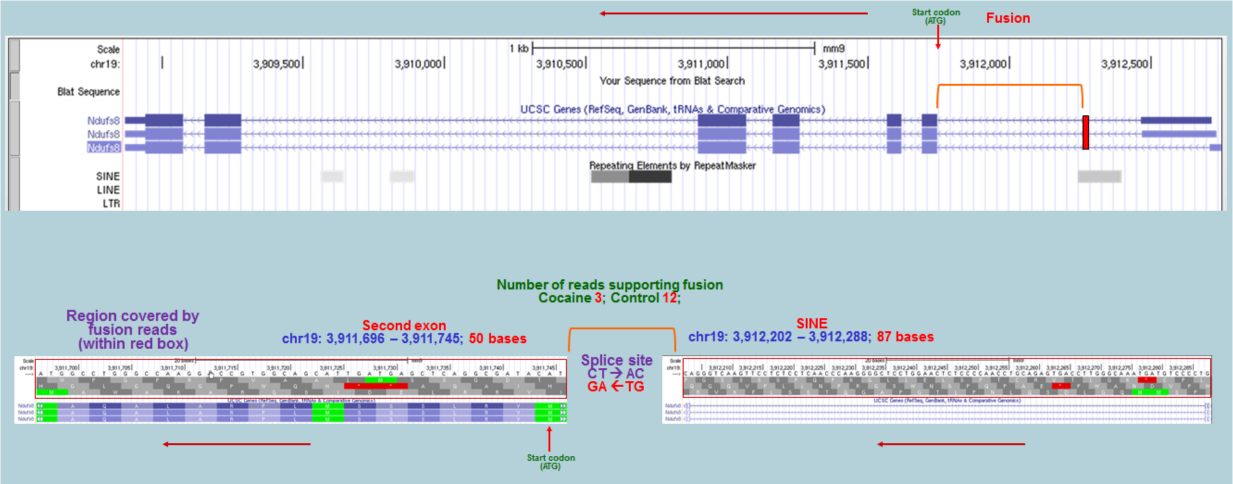

Orc3 fusions with SINE

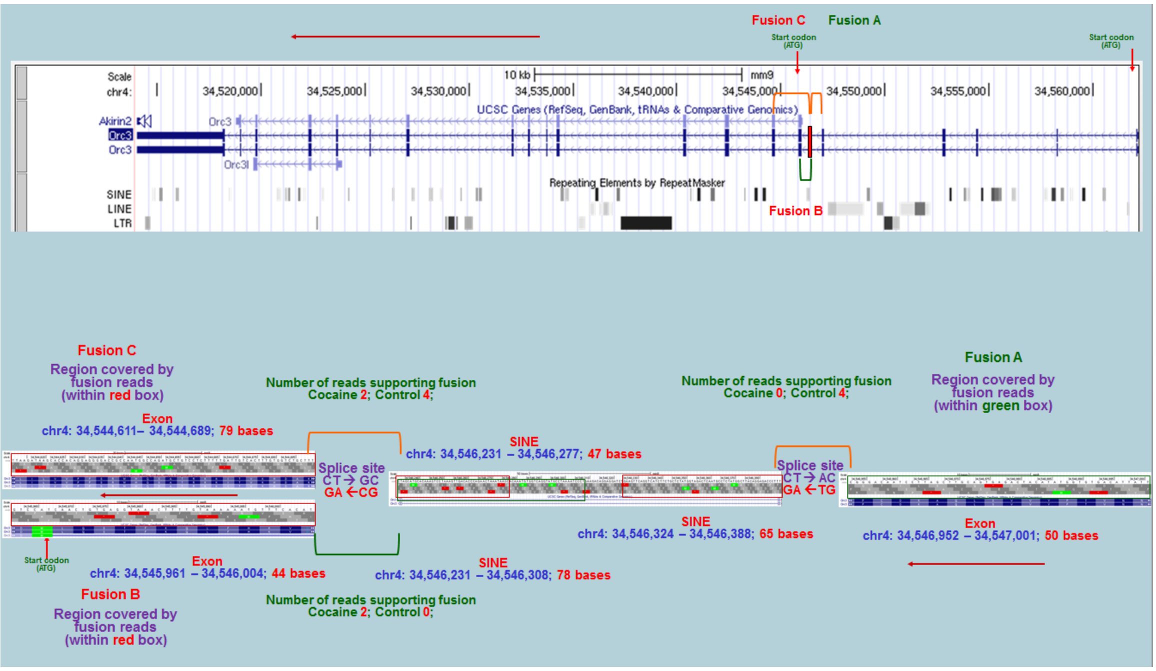

Slc43a2 fusion with MER1 DNA repeat

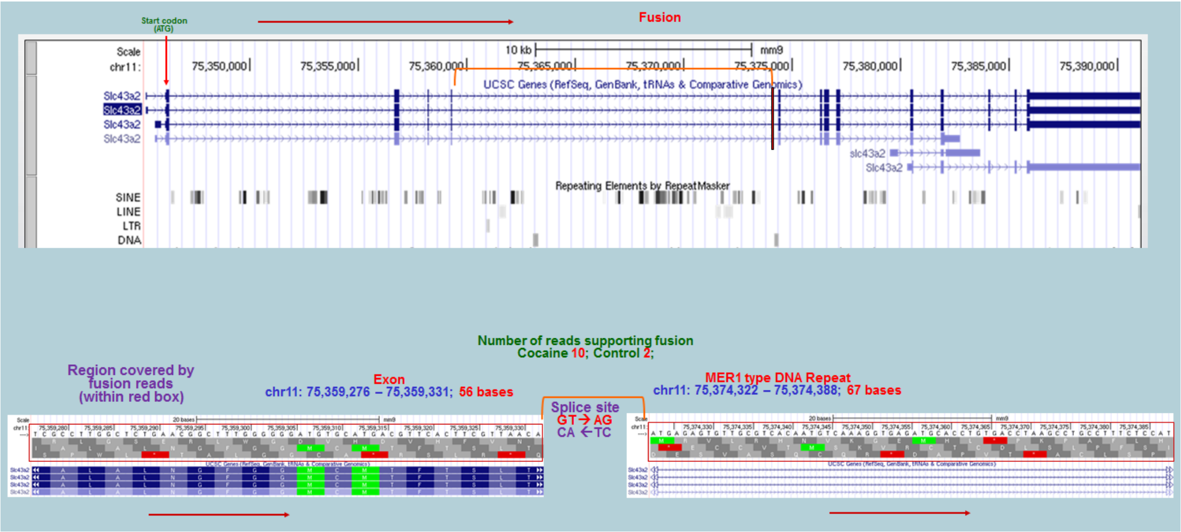

Srrm2 fusion with SINE

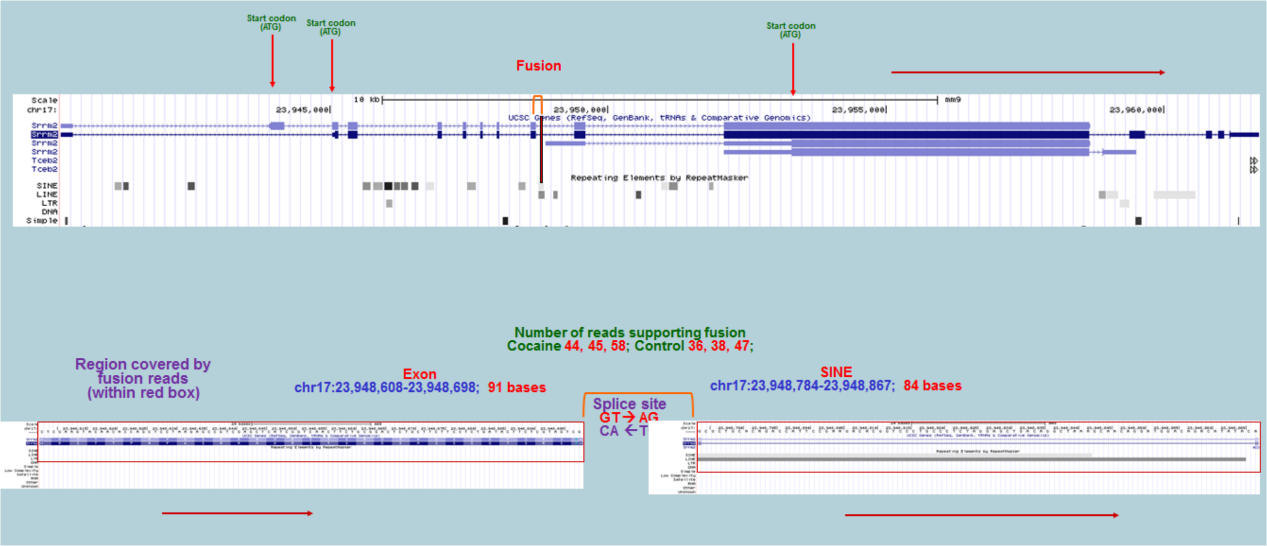

Timm44 fusions with SINE

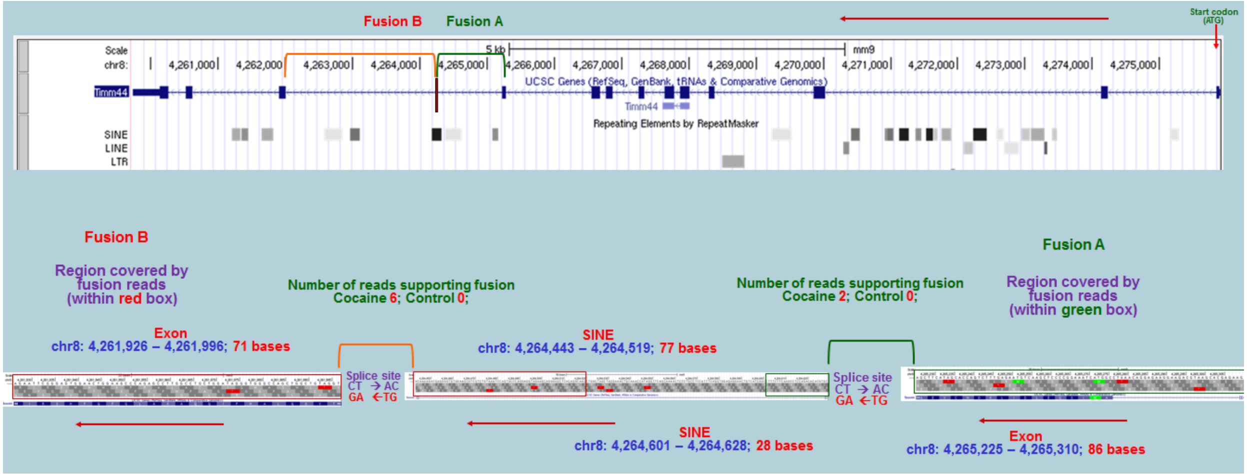

Supplement: S5 Fig — Representation of the fusion events predicted in 24 additional randomly selected genes out of the 813 events detected. The canonical start codon (ATG) is shown based on gene annotation; directionally of transcription is indicated with red arrow above and below the panels based on UCSC gene annotation. In all cases, top panel shows full view of gene structure. TSS indicates the transcription start site. Middle and/or lower panels show enlarged view of different predicted fusions, including the splicing recognition sites (GT-AG). Orange or green brackets indicate regions involved in the fusion (repeat/exon or vice-versa). Red boxes indicate the region from which RNA-seq reads support the fusion events; when more than one fusion was identified on the same gene (isoform) green boxes identify the reads supporting the additional event. The number of reads supporting the fusion event is also depicted. (PDF) [file pone.0159028.s005.pdf]
